# Supplementary material for: The impact of climate change induced alterations of streamflow and stream temperature on the distribution of riparian species
Source: PLoS One. 2020 Nov 24;15(11):e0242682. doi: 10.1371/journal.pone.0242682 (PMC7685490; doi:10.1371/journal.pone.0242682)
Supplement: S1 File — (DOCX) [file pone.0242682.s001.docx]

## S1 File

Spatially explicit probabilities of species occurrence and relationships of species occurrence and environmental metrics are available for viewing and download at https://sccwrp.shinyapps.io/flowecology/.

Additional information for the species clustering, model performance, and results: variables used in species clustering **(**S-1), clustering analysis results (S-2), sources used for the species observation data (S-3), streamflow and temperature metrics (S-4), streamflow metrics and species occurrence simple logistic regression results (S-5), performance of the random forest model of species occurrence using streamflow (S-6), results of the principal components analysis (S-7), and temperature metric relationships with species probability of occurrence for help interpreting the logistic regression results (S-8).

### **S-1: Variables used in clustering analysis**

Table S-1: Variables used for grouping species

| Variable | Categories |
| --- | --- |
| General habitat | Main channel, backwater, riparian, wetland, variable |
| Foraging behavior | Dabble, dive, fly, run, stalk, swim |
| Vegetation preference | Aquatic, overhanging, scrub, woodland, none |
| Prey preference (birds only) | Fruit, seed, grain, plant, fish, bird/mammal, terrestrial invertebrate, aerial invertebrate, aquatic invertebrate, amphibian |
| Water velocity | Fast, medium, slow, NA |
| Preferred substrate | Fine, sandy/gravel, cobble, boulder, NA |
| Nest location | Submerged substrate, emergent vegetation, nest at the bottom of a channel, cavity within a channel, ground, tree, shrub, bank, variable, NA |
| Stream category | Permanent, temporary, NA |
| Stream depth (fish and herps only) | Shallow, average, deep |
| Stream temperature (fish and herps only) | Cool, warm, hot |

### **S-2:** Species clusters

Table S-2: Riparian species (vertebrates) in the study area that were included in the clustering analysis. The last column, ‘Cluster’, shows the grouping. The bolded rows are the species selected as the focal species.

| Common name | Name | Group | Sensitive | Native | Cluster |
| --- | --- | --- | --- | --- | --- |
| African clawed frog | *Xenopus laevis* | Amphibian |  |  | 1 |
| American bullfrog | *Lithobates catesbeianus* | Amphibian |  | Y | 1 |
| Baja California treefrog | *Pseudacris hypochondriaca hypochondriaca* | Amphibian |  | Y | 1 |
| Western spadefoot | *Spea hammondii* | Amphibian | Y | Y | 1 |
| Red-eared slider | *Trachemys scripta elegans* | Reptile |  |  | 1 |
| Snapping turtle | *Chelydra serpentina* | Reptile |  |  | 1 |
| Western painted turtle | *Chrysemys picta bellii* | Reptile |  |  | 1 |
| Fathead minnow | *Pimephales promelas* | Fish |  |  | 1 |
| Golden shiner | *Notemigonus crysoleucas* | Fish |  |  | 1 |
| Western Mosquitofish | *Gambusia affinis* | Fish |  |  | 1 |
| Red shiner | *Cyprinella lutrensis* | Fish |  |  | 1 |
| Green Sunfish | *Lepomis cyanellus* | Fish |  |  | 1 |
| Two-striped garter snake | *Thamnophis hammondii* | Reptile |  | Y | 2 |
| Texas spiny softshell | *Apalone spinifera emoryi* | Reptile |  |  | 2 |
| **Arroyo chub** | ***Gila orcuttii*** | **Fish** | **Y** | **Y** | **2** |
| Threespine stickleback | *Gasterosteus aculeatus* | Fish | Y | Y | 2 |
| California treefrog | *Pseudacris cadaverina* | Amphibian |  | Y | 3 |
| California toad | *Anaxyrus boreas halophilus* | Amphibian |  | Y | 3 |
| **Western pond turtle** | ***Actinemys marmorata*** | **Reptile** |  | **Y** | **3** |
| **Santa Ana sucker** | ***Catostomus santaanae*** | **Fish** | **Y** | **Y** | **3** |
| California newt | *Taricha torosa* | Amphibian | Y | Y | 3 |
| California red-legged frog | *Rana draytonii* | Amphibian | Y | Y | 3 |
| Black bullhead | *Ameiurus melas* | Fish |  |  | 4 |
| Bluegill sunfish | *Lepomis macrochirus* | Fish |  |  | 4 |
| Brown bullhead | *Ameiurus nebulosus* | Fish |  |  | 4 |
| Largemouth bass | *Micropterus salmoides* | Fish |  |  | 4 |
| Tilapia spp | *Oreochromis* | Fish |  |  | 4 |
| Yellow bullhead | *Ameiurus natalis* | Fish |  |  | 4 |
| Common carp | *Cyprinus carpio* | Fish |  |  | 4 |
| Brown trout | *Salmo trutta* | Fish |  |  | 5 |
| Channel catfish | *Ictalurus punctatus* | Fish |  |  | 5 |
| **Coastal rainbow trout / steelhead** | ***Oncorhynchus mykiss irideus*** | **Fish** | **Y** | **Y** | **5** |
| Santa Ana speckled dace | *Rhinichthys osculus* | Fish | Y | Y | 5 |
| Mountain yellow-legged frog | *Rana muscosa* | Amphibian | Y | Y | 5 |
| **Arroyo toad** | ***Anaxyrus californicus*** | **Amphibian** | **Y** | **Y** | **6** |
| California least tern | *Sterna antillarum browni* | Bird | Y | Y | 7 |
| Spotted sandpiper | *Actitis macularius* | Bird |  | Y | 7 |
| Black-necked stilt | *Himantopus mexicanus* | Bird |  | Y | 7 |
| Bank swallow | *Riparia riparia* | Bird | Y | Y | 8 |
| Common yellowthroat | *Geothlypis trichas* | Bird |  | Y | 8 |
| **Least Bell's vireo** | ***Vireo bellii pusillus*** | **Bird** | **Y** | **Y** | **8** |
| Lincoln's sparrow | *Melospiza lincolnii* | Bird |  | Y | 8 |
| MacGillivray's warbler | *Geothlypis tolmiei* | Bird |  | Y | 8 |
| Swainson's thrush | *Catharus ustulatus* | Bird |  | Y | 8 |
| Willow flycatcher | *Empidonax traillii* | Bird | Y | Y | 8 |
| Wilson's warbler | *Cardellina pusilla* | Bird |  | Y | 8 |
| Yellow warbler | *Setophaga petechia* | Bird |  | Y | 8 |
| Yellow-billed cuckoo | *Coccyzus americanus* | Bird | Y | Y | 8 |
| Yellow-breasted chat | *Icteria virens* | Bird | Y | Y | 8 |
| Black-crowned night heron | *Nycticorax nycticorax* | Bird |  | Y | 9 |
| Great blue heron | *Ardea herodias* | Bird |  | Y | 9 |
| Great egret | *Ardea alba* | Bird |  | Y | 9 |
| Green heron | *Butorides virescens* | Bird |  | Y | 9 |
| Pied-billed grebe | *Podilymbus podiceps* | Bird |  | Y | 9 |
| Snowy egret | *Egretta thula* | Bird |  | Y | 9 |
| Wilson's snipe | *Gillinago delicata* | Bird |  | Y | 9 |
| Cinnamon teal | *Anas cyanoptera* | Bird |  | Y | 10 |
| Mallard | *Anas platyrhynchos* | Bird |  | Y | 10 |
| Northern pintail | *Anas acuta* | Bird |  | Y | 10 |
| Song sparrow | *Melospiza melodia* | Bird |  | Y | 10 |
| Wood Duck | *Aix sponsa* | Bird |  | Y | 10 |
| Brown-headed cow bird | *Molothrus ater* | Bird |  | Y | 10 |
| Cooper's hawk | *Accipiter cooperii* | Bird |  | Y | 11 |
| Long-eared owl | *Asio otus* | Bird |  | Y | 11 |
| Red-shouldered hawk | *Buteo lineatus* | Bird |  | Y | 11 |
| American dipper | *Cinclus mexicanus* | Bird |  | Y | 12 |

### **S-3:** Sources used for compiling the species distribution data

BonTerra Consulting. 2012. Results of Focused Presence/Absence Least Bell’s Vireo and Southwestern Willow Flycatcher Surveys for the Big Tujunga Dam and Reservoir Sediment Removal Project, Los Angeles County, California. Email to Ms. Susie Tharratt, Recovery Permit Coordinator, Carlsbad Fish and Wildlife Office.

BonTerra Consulting. 2013. Results of the 2013 Least Bell’s Vireo and Southwestern Willow Flycatcher Surveys for the Arroyo Seco Canyon Project in the City of Pasadena, Los Angeles County, California. Email to Mr. David Rydman, Carollo Engineers, Inc.

BonTerra Psomas. 2017. 2017 Focused Survey Results. Los Angeles County Flood Control District Soft-Bottom Channels Maintenance Clearing. Report prepared for Los Angeles County Flood Control District; Flood Maintenance Division.

California Department of Fish and Wildlife. 2013 – 2017. Field observations of special status and novel species in the Ventura River basin and Sisar and Santa Paula Creek (Santa Clara River Basin). Data provided by Mary Larson, Steelhead Restoration and Recovery Unit, California Department of Fish and Wildlife.

Dagit, Rosi. 2016. Field Notes. Sepulveda Dam – Los Angeles River. Fish Survey for FOLAR, November 22, 2016. Research Conservation District of the Santa Monica Mountains.

ECORP Consulting, Inc. 2010. Report for the Santa Ana Sucker (*Catostomus santaanae*) Survey and Relocation Effort in the Big Tujunga Wash at Oro Vista Avenue (W.O. E1907366). Report prepared for the City of Los Angeles. Submitted by EnviCraft LLC.

Environmental Science Associates. 2014. Middle Piru Creek 2014 Arroyo Toad (Anaxyrus californicus) Clutch Surveys and Sensitive Species Monitoring. Report prepared for California Department of Water Resources.

Environmental Science Associates. 2015. Middle Piru Creek 2015 Arroyo Toad (Anaxyrus californicus) Clutch Surveys and Sensitive Species Monitoring. Report prepared for California Department of Water Resources.

Environmental Science Associates. 2016. Middle Piru Creek 2016 Arroyo Toad (Anaxyrus californicus) Clutch Surveys and Sensitive Species Monitoring. Report prepared for California Department of Water Resources.

Environmental Science Associates. 2017. Middle Piru Creek 2017 Arroyo Toad (Anaxyrus californicus) Clutch Surveys and Sensitive Species Monitoring. Report prepared for California Department of Water Resources.

Guthrie, Daniel A. 1999. Bird Surveys Along the Santa Clara River, 1999. Ventura County line Downstream to Just Below Las Brisas Crossing. W. M. Keck Science Center, Claremont Colleges. Report prepared for Newhall Land and Farming.

Guthrie, Daniel A. 2000. Bird Surveys Along a Portion of the Santa Clara River and its Tributaries Upstream from the Castaic Creek Confluence, Near Valencia, California, 2000. W. M. Keck Science Center, Claremont Colleges. Report prepared for the Valencia Corporation.

Guthrie, Daniel A. 2001. Bird Surveys Along A portion of the Santa Clara River and its Tributaries Upstream from the Castaic Creek Confluence near Valencia California, 2001. W. M. Keck Science Center, Claremont Colleges. Report prepared for the Valencia Corporation.

Guthrie, Daniel A. 2002. Bird Surveys along the Santa Clara River, 2002, Mouth of Castaic Creek Downstream to Just Below Las Brisas Crossing. W. M. Keck Science Center, Claremont Colleges. Report prepared for the Valencia Corporation.

Guthrie, Daniel A. 2003. Bird Surveys Along a Portion of the Santa Clara River and its Tributaries Upstream from the Castaic Creek Confluence near Valencia, California, 2003. W. M. Keck Science Center, Claremont Colleges. Report prepared for the Valencia Corporation.

Guthrie, Daniel A. 2003. Bird Surveys Along the Santa Clara River, 2003, Mouth of Castaic Creek Downstream to just Below Las Brisas Crossing. W. M. Keck Science Center, Claremont Colleges. Report prepared for the Valencia Corporation.

Guthrie, Daniel A. 2004. Bird Surveys along the Santa Clara River, 2004, Mouth of Castaic Creek Downstream to just Below Las Brisas Crossing. W. M. Keck Science Center, Claremont Colleges. Report prepared for the Valencia Corporation.

Guthrie, Daniel A. 2005. Bird Surveys Along a Portion of the Santa Clara River and its Tributaries Upstream from the Castaic Creek Confluence, near Valencia, California, 2005. W. M. Keck Science Center, Claremont Colleges. Report prepared for the Valencia Corporation.

Guthrie, Daniel A. 2005. Bird Surveys along the Santa Clara River, 2005, Mouth of Castaic Creek Downstream to just Below Las Brisas Crossing. W. M. Keck Science Center, Claremont Colleges. Report prepared for the Valencia Corporation.

Guthrie, Daniel A. 2006. Bird Surveys along the Santa Clara River, 2006, Mouth of Castaic Creek Downstream to just Below Las Brisas Crossing. W. M. Keck Science Center, Claremont Colleges. Report prepared for the Valencia Corporation.

Haglund, Thomas R. & Baskin, Jonathan N. 1995. Sensitive Aquatic Species Survey. Santa Clara River and San Francisquito Creek. Newhall Land and Farming Company Property. Los Angeles County, California. San Marino Environmental Associates.

Haglund, Thomas R. & Baskin, Jonathan N. 2000. Fish and Wildlife Survey and Habitat Assessment of the Santa Clara River at Interstate 5. California State Polytechnic University, Pomona.

Haglund, Thomas R. & Baskin, Jonathan N. 2005. Tesoro Stickleback Survey Memorandum. San Francisquito Creek. San Marino Environmental Associates.

Haglund, Thomas R. & Baskin, Jonathan N. 2005. Tapia Canyon Road Fish Survey Memorandum. Castaic Creek. San Marino Environmental Associates.

Haglund, Thomas R. & Baskin, Jonathan N. 2006. Big Tujunga Wash Project Memorandum. Big Tujunga Creek. San Marino Environmental Associates.

Haglund, Thomas R. & Baskin, Jonathan N. No Date. Distribution and Anatomy of Threespine Sticklebacks in the Santa Clara River, California, 2007-2010. San Marino Environmental Associates.

San Marino Environmental Associates. 1994. Southwestern Pond Turtle Data. ARCO Natural Resource Damage Assessment.

Haglund, Thomas R. & Baskin, Jonathan N. 2004. Habitat Conservation Plan for the Federally Endangered Unarmored Threespine Stickleback and Other Species of Special Concern at the Newhall Land and Farming Company's Crossings of the Santa Clara River, Los Angeles and Ventura Counties, California. San Marino Environmental Associates.

Hofflander, Dylan & Dagit, Rosi. 2015. Field Notes. Sepulveda Dam - Los Angeles River. Fish Survey for FOLAR, November 23, 2015. Watershed Steward and Research Conservation District of the Santa Monica Mountains.

Howard, Steve & Gray, Sara. 2008. Fish Passage Monitoring and Studies; Vern Freeman Diversion Facility; Santa Clara River, Ventura County, California. Annual Report. 2008 Monitoring Season. Report prepared for United Water Conservation District, Santa Paula, California.

Howard, Steve & Gray, Sara. 2009. Fish Passage Monitoring and Studies; Vern Freeman Diversion Facility; Santa Clara River, Ventura County, California. Annual Report. 2009 Monitoring Season. Report prepared for United Water Conservation District, Santa Paula, California.

Howard, Steve & Gray, Sara. 2010. Fish Passage Monitoring and Studies; Vern Freeman Diversion Facility; Santa Clara River, Ventura County, California. Annual Report. 2010 Monitoring Season. Report prepared for United Water Conservation District, Santa Paula, California.

Howard, Steve & Booth, Mike. 2011. Fish Passage Monitoring and Studies; Vern Freeman Diversion Facility; Santa Clara River, Ventura County, California. Annual Report. 2011 Monitoring Season. Report prepared for United Water Conservation District, Santa Paula, California.

Howard, Steve & Booth, Mike. 2012. Fish Passage Monitoring and Studies; Freeman Diversion Facility; Santa Clara River, Ventura County, California. Annual Report. 2012 Monitoring Season. Report prepared for United Water Conservation District, Santa Paula, California.

Howard, Steve & Booth, Mike. 2012. Fish Passage Monitoring and Studies; Freeman Diversion Facility; Santa Clara River, Ventura County, California. Annual Report. 2012 Monitoring Season. Report prepared for United Water Conservation District, Santa Paula, California.

Howard, Steve & Booth, Mike. 2013. Fish Passage Monitoring and Studies; Freeman Diversion Facility; Santa Clara River, Ventura County, California. Annual Report. 2013 Monitoring Season. Report prepared for United Water Conservation District, Santa Paula, California.

Howard, Steve & Booth, Mike. 2014. Fish Passage Monitoring and Studies; Freeman Diversion Facility; Santa Clara River, Ventura County, California. Annual Report. 2014 Monitoring Season. Report prepared for United Water Conservation District, Santa Paula, California.

Howard, Steve. & Jacinto, Monica. 2018. Arroyo Toad Clutch Surveys. Sespe Creek- Beaver Campground Reach. Summary Report, 2017. Report prepared for United States Fish and Wildlife Service and United States Geological Survey.

Impact Sciences, Inc. 2003. Results of Focused Surveys for Unarmored Threespine Stickleback and Other Special-Status Fish Species; Newhall Ranch, Valencia, California. Report prepared for Newhall Land and Farming.

Impact Sciences, Inc. 2014. Results of Focused Surveys for Arroyo Toad and Special-Status Herpetofauna. Mission Village Project. Newhall Ranch. Report prepared for Newhall Land and Farming Company.

Impact Sciences, Inc. & UltraSystems Environmental, Inc. 2014. Results of Focused Arroyo Toad Surveys; Pine Canyon Road Improvement Project; Lake Hughes, CA.

Matthews, K. R. & Berg, N. H. 1997. Rainbow Trout Responses to Water Temperature and Dissolved Oxygen Stress in two Southern California Stream Pools. Journal of Fish Biology, 50, 50-67.

Occurrence Information for Multiple Species within Jurisdiction of the Carlsbad Fish and Wildlife Office (CFWO). U.S. Fish and Wildlife Service, Carlsbad Fish and Wildlife Office. Download available at <https://www.fws.gov/carlsbad/GIS/CFWOGIS.html>.

Research Conservation District of the Santa Monica Mountains. 2001-2018. Presence or Absence of Steelhead/Resident O. mykiss. Santa Monica Coastal Creeks. Data provided by Rosi Dagit.

Resource Conservation District of the Santa Monica Mountains. 2008 – 2016. Field observations of Steelhead (O. mykiss) in Malibu Creek and Topanga Creek in the Santa Monica Mountains. Data provided by Mary Larson.

Research Conservation District of the Santa Monica Mountains. 2018. Species occurrence data collected for submission to CNDDB. Santa Monica Coastal Creeks. Data provided by Rosi Dagit.

Sasaki, Shoken. 1986. California Wild Trout Management Program. Sespe Creek Wild Trout Management Plan. Sespe Creek, Ventura County. California Department of Fish and Game (now, CA Dept of Fish and Wildlife).

Stoecker, M. and E. Kelley. 2005. Santa Clara River Steelhead Trout: Assessment and Recovery Opportunities. Report prepared for The Nature Conservancy and The Santa Clara River Trustee Council. pp. 294.

United States Department of Fish and Wildlife. Fish Data Base. Data provided by John Baskin.

Weaver, Jeff & Mehalick, Stephanie. 2008. Fish Creek and Agua Blanca Creek Summary Report. June 16-19th, 2008. Heritage and Wild Trout Program. California Department of Fish and Game (now, CA Dept of Fish and Wildlife).

Weaver, Jeff & Mehalick, Stephanie. 2008. Upper Piru Creek Summary Report. Snowy, Buck, Piru, Alamo, and Mutau Creeks. June 11-13, 2008. Heritage and Wild Trout Program. California Department of Fish and Game (now, CA Dept of Fish and Wildlife).

Weaver, Jeff & Mehalick, Stephanie. 2009. East Fork San Gabriel River 2009 Summary Report. June 23-25, 2009. State of California. Natural Resources Agency. Heritage and Wild Trout Program. California Department of Fish and Game (now, CA Dept of Fish and Wildlife).

Weaver, Jeff & Mehalick, Stephanie. 2010. East Fork San Gabriel River 2010 Summary Report. August 26-31, 2010. State of California. Natural Resources Agency. Heritage and Wild Trout Program. California Department of Fish and Game (now, CA Dept of Fish and Wildlife).

### **S-4:** Streamflow and temperature metrics.

Table S-4: Streamflow and temperature metrics. Script used for calculation and definition came from Konrad et al., 2008 except for the last three variables in the table which we calculated separately. Timeframe refers to the number of years of flow data used in the calculation, measured back in time, from the year of analysis (either the year the species was observed or the wet/dry/moderate year of interest). For temperature, the months included in calculating these metrics are May through September.

| Variable | Pattern | Definition [units] | Timeframe |
| --- | --- | --- | --- |
| Streamflow | | | |
| Qmean | Magnitude | [m^3^/s] mean Q for the period of analysis | 3, 5 10, all |
| QmeanMedian | Magnitude | [m^3^/s] median annual mean Q | 3, 5 10, all |
| Qmax | Magnitude | [m^3^/s] median annual maximum daily Q | 3, 5 10, all |
| Qmin | Magnitude | [m^3^/s] median annual minimum daily Q | 3, 5 10, all |
| QmeanIDR | Variability | [m^3^/s] Interdecile range of mean Q | 3, 5 10, all |
| QmaxIDR | Variability | [m^3^/s] Interdecile range of maximum Q | 3, 5 10, all |
| QminIDR | Variability | [m^3^/s] Interdecile range of minimum Q | 3, 5 10, all |
| Qmed | Magnitude | [m^3^/s] median daily Q | 3, 5 10, all |
| HighNum | Frequency | [events/year] number of events > high flow threshold. | 3, 5 10, all |
| LowNum | Frequency | [events/year] number of events <= low flow threshold. | 3, 5 10, all |
| HighDur | Duration | [days/event] - longest consecutive days > the high flow threshold | 3, 5 10, all |
| LowDur | Duration | [days/event]- longest consecutive days <= the low flow threshold | 3, 5 10, all |
| NoDisturb | Duration | [days] - longest number of consecutive days between the low and high flow threshold | 3, 5 10, all |
| Hydroperiod | Duration | [% of years] - fraction of period of analysis with Q | 3, 5 10, all |
| FracYearsNoFlow | Frequency | [% of years] - fraction of years with at least one no-flow day | 3, 5 10, all |
| Mednoflowdays | Frequency | [days/year]- median annual number of no-flow days | 3, 5 10, all |
| RecessMaxLength | Duration | [days] Maximum length of Q recession | 3, 5 10, all |
| R10D.5 | Variability | [%/day] - Median 10-day recession rate for low flow year | 3, 5 10, all |
| R10D.9 | Variability | [%/day] - 90% percentile 10-day recession rate for low flow year | 3, 5 10, all |
| R10D4D | Variability | [%/day] - 10-day recession rate starting after 4 days of recession | 3, 5 10, all |
| BFR | Variability | [%/day] - Base flow recession. | 3, 5 10, all |
| SFR | Variability | [%/day] - Storm flow recession. | 3, 5 10, all |
| MaxMonth | Timing | [1= Jan] - month of maximum mean monthly Q | 3, 5 10, all |
| MinMonth | Timing | [1= Jan] - month of minimum mean monthly Q | 3, 5 10, all |
| Max Month Q | Magnitude | [m^3^/s] - maximum mean monthly Q | 3, 5 10, all |
| Min Month Q | Magnitude | [m^3^/s] - minimum mean monthly Q | 3, 5 10, all |
| Q01-Q99 | Magnitude | [m^3^/s] - Q quantiles | 3, 5 10, all |
| Oct - Sept | Magnitude | [m^3^/s] - Mean Q in the 12 months preceding a specific date | 1 |
| RBI | Variability | [unitless] Richards-Baker flashiness Index. | 3, 5 and 10 |
| Twoyr, fivyr, tenyr | Timing | [days] - Number of days from a specific date to a storm. | all |
| Temperature | | | |
| Minimum 7-day minimum | Magnitude | [°C] The minimum value of a rolling 7-day minimum | 1 |
| Maximum 7-day maximum | Magnitude | [°C] The maximum value of a rolling 7-day maximum | 1 |
| Maximum 7-day average | Magnitude | [°C] The maximum value of a rolling 7-day average | 1 |
| Maximum 7-day range | Variability | [°C] The maximum difference between the rolling 7-day maximum and minimum. I.e. the largest temperature swing within a 7-day period. | 1 |
| Mean 7-day range | Variability | [°C] The average difference between the rolling 7-day maximum and minimum. I.e. the average temperature swing within a 7-day period. | 1 |
| Number of 7-day maximums > 30°C | Frequency | [days] The number of 7-day rolling averages that are greater than 30°C | 1 |

### **S-5:** Streamflow metrics and focal species presence or absence relationship simple logistic regression results

Table S-5: Univariate logistic regression results for species presence or absence and a subset of the streamflow metrics. Sign refers to the direction of the coefficient. Significance codes: p<0.001 ***; p<0.01 **; p<0.05 * except for *A. californicus* where P<0.2 *. The time refers to the length of streamflow timeseries used to calculate the flow metric. All = the entire streamflow timeseries; 3, 5, 10 = three, five, and ten years of flow data terminating on the date of species occurrence record. Variables defined in S-4.

| Time | Flow Metric | *O. mykiss irideus* | *G. orcuttii* | *A. californicus* | *V. bellii pusillus* | *C. santaanae* | *A. marmorata* |
| --- | --- | --- | --- | --- | --- | --- | --- |
| Flashiness | | | | | | | |
| 10 | BFR | 24 *** | -8.96 *** | -45.83 * | -17.57 *** | -7.99 *** | NA |
| 5 |  | 8.55 *** | -6.29 ** | -40.89 * | -15.04 *** | -5.05 ** | NA |
| 3 |  | 10.41 *** | -8.04 *** | -52 * | -14.59 *** | -7.19 *** | NA |
| all |  | 24.43 *** | -8.23 *** | -26.59 NS | -17.12 *** | -6.68 *** | -1.6 NS |
| 10 | R10D.5 | 7.1 *** | -13.07 *** | -3.88 * | -7.97 *** | -11.86 *** | NA |
| 5 |  | 6.66 *** | -11.02 *** | -2.73 NS | -7.11 *** | -10.22 *** | NA |
| 3 |  | 6.58 *** | -13.84 *** | -2.33 NS | -7.7 *** | -12.15 *** | NA |
| all |  | 7.21 *** | -10.67 *** | -2.3 NS | -5.72 *** | -10.4 *** | -1.66 NS |
| 10 | R10D.9 | 6.9 *** | -9.41 *** | -10.33 * | -5.95 *** | -8.64 *** | NA |
| 5 |  | 5.92 *** | -9.56 *** | -6.45 * | -5.36 *** | -8.9 *** | NA |
| 3 |  | 6.66 *** | -10.38 *** | -4.94 * | -6.17 *** | -9.34 *** | NA |
| all |  | 6.72 *** | -8.25 *** | -17.26 NS | -5.38 *** | -7.71 *** | -5.13 *** |
| 10 | R10D4D | 5.97 *** | -1.28 NS | 0.79 NS | -9.48 *** | -0.74 NS | NA |
| 5 |  | 3.76 *** | -1.51 NS | 0.3 NS | -9.82 *** | -0.32 NS | NA |
| 3 |  | 4.91 *** | -5.29 *** | -2.07 NS | -10.96 *** | -5.09 *** | NA |
| all |  | 5.84 *** | -1.28 NS | 1.83 NS | -8.38 *** | -0.61 NS | -1.25 NS |
| 10 | SFR | 8.33 *** | -5.63 *** | -5.97 NS | -6.4 *** | -5.25 *** | NA |
| 5 |  | 5.74 *** | -5.27 *** | -6.66 * | -6.41 *** | -4.85 *** | NA |
| 3 |  | 5.86 *** | -5.69 *** | -9.48 * | -6.93 *** | -5.46 *** | NA |
| all |  | 6.59 *** | -5.25 *** | -2.82 NS | -6.1 *** | -4.77 *** | 0.41 NS |
| 10 | RBI | -0.52 NS | 4.2 *** | -0.15 NS | 6.31 *** | 4.14 *** | NA |
| 5 |  | 0.78 NS | 4.98 *** | 2.71 NS | 6.14 *** | 4.11 *** | NA |
| 3 |  | -1.16 * | 4.47 *** | 3.94 * | 6.07 *** | 6.81 *** | NA |
| all |  | -2.31 *** | 4.52 *** | 2.6 NS | 7.18 *** | 5.38 *** | 3.28 *** |
| Duration | | | | | | | |
| 10 | Frac Years No Flow | -2.85 *** | 2.14 *** | 3.56 NS | 8.67 *** | 2.56 *** | NA |
| 5 |  | -2.92 *** | 2.39 *** | 3.71 * | 6.69 *** | 2.69 *** | NA |
| 3 |  | -2.79 *** | 2.5 *** | 3.61 * | 6.07 *** | 2.82 *** | NA |
| all |  | -2.68 *** | 1.82 *** | -33.03 * | 8.3 *** | 2.3 *** | 1957.87 NS |
| 10 | HighDur | 0.01 *** | -0.01 * | -0.01 NS | -0.02 NS | 0 *** | NA |
| 5 |  | 0.01 *** | -0.01 ** | 0.04 NS | 0.01 NS | 0 *** | NA |
| 3 |  | 0.02 *** | -0.01 * | 0 NS | 0.02 NS | 0.01 *** | NA |
| all |  | 0.28 *** | -0.13 NS | 0.05 NS | -0.06 NS | -0.14 NS | -1.49 ** |
| 10 | Hydroperiod | 3.32 *** | -2.83 *** | 4.19 NS | -8.51 *** | -3.21 *** | NA |
| 5 |  | 3.18 *** | -2.85 *** | -0.94 NS | -7.91 *** | -3.11 *** | NA |
| 3 |  | 3.14 *** | -2.73 *** | -2.38 NS | -6.42 *** | -2.97 *** | NA |
| all |  | 3.3 *** | -2.47 *** | 13.81 * | -8.11 *** | -2.8 *** | -4325.92 NS |
| 10 | LowDur | 0 *** | 0 NS | -0.01 NS | 0.02 ** | 0 NS | NA |
| 5 |  | 0 *** | 0 NS | 0 NS | 0.01 *** | 0 NS | NA |
| 3 |  | 0 *** | 0 NS | 0 NS | 0.01 *** | 0 * | NA |
| all |  | 0 *** | 0 ** | 0.02 NS | 0.03 ** | 0 ** | 0.01 ** |
| 10 | Median No Flow Days | -0.01 *** | 0.01 *** | -0.03 NS | 0.02 *** | 0.01 *** | NA |
| 5 |  | -0.01 *** | 0.01 *** | 0 NS | 0.02 *** | 0.01 *** | NA |
| 3 |  | -0.01 *** | 0.01 *** | 0.01 NS | 0.02 *** | 0.01 *** | NA |
| all |  | -0.01 *** | 0.01 *** | -0.12 * | 0.02 *** | 0.01 *** | 19.86 NS |
| 10 | NoDisturb | 0 *** | 0 NS | 0 NS | -0.02 *** | -0.01 *** | NA |
| 5 |  | 0.01 *** | -0.01 ** | -0.01 * | -0.02 *** | -0.02 *** | NA |
| 3 |  | 0 *** | 0 *** | 0 NS | -0.02 *** | -0.01 *** | NA |
| all |  | 0 *** | -0.01 *** | 0 NS | -0.01 *** | -0.01 *** | 0 * |
| 10 | Recess Max Length | 0 *** | -0.01 *** | 0 NS | -0.01 *** | -0.01 *** | NA |
| 5 |  | 0 *** | -0.01 *** | 0 NS | -0.01 *** | -0.01 *** | NA |
| 3 |  | 0 *** | 0 *** | 0 * | -0.01 ** | -0.01 *** | NA |
| all |  | 0 *** | -0.01 *** | 0 NS | -0.01 *** | -0.01 *** | 0.02 * |
| Frequency | | | | | | | |
| 10 | High Num | -0.43 *** | 0.4 *** | 0.04 NS | 0.4 *** | 0.34 *** | NA |
| 5 |  | -0.22 ** | 0.53 *** | 0.26 NS | 0.47 *** | 0.47 *** | NA |
| 3 |  | -0.19 ** | 0.54 *** | 0.23 NS | 0.51 *** | 0.5 *** | NA |
| all |  | -0.3 ** | 0.39 *** | 0.12 NS | 0.43 *** | 0.36 *** | -0.23 *** |
| 10 | Low Num | -0.38 *** | 0.15 *** | 0.11 NS | 0.18 *** | 0.15 *** | NA |
| 5 |  | -0.33 *** | 0.2 *** | 0.27 NS | 0.22 *** | 0.19 *** | NA |
| 3 |  | -0.36 *** | 0.21 *** | -0.1 NS | 0.23 *** | 0.2 *** | NA |
| all |  | -0.42 ** | 0.16 *** | 0 NS | 0.18 *** | 0.16 *** | -0.02 NS |
| Timing | | | | | | | |
| 10 | Max Month | 1.04 *** | -0.56 NS | 0.06 NS | -1.1 * | -2.86 *** | NA |
| 5 |  | -0.01 NS | -0.11 NS | 0.07 NS | -0.93 NS | -0.84 *** | NA |
| 3 |  | 0.07 NS | -0.28 ** | -0.2 * | -1.34 *** | -0.96 *** | NA |
| all |  | 1.63 *** | -0.15 NS | 0 NS | -182.82 NS | 0.42 NS | -0.54 NS |
| 10 | Min Month | 0.26 *** | -0.23 ** | -0.85 * | -1.54 *** | -0.83 *** | NA |
| 5 |  | 0.62 *** | -0.05 NS | 0.08 NS | -1.27 *** | -0.07 NS | NA |
| 3 |  | 0.59 *** | -0.05 NS | 0.11 NS | -1.22 *** | -0.19 NS | NA |
| all |  | 0.23 *** | -0.28 *** | -0.61 NS | -1.33 *** | -0.42 *** | -0.14 ** |
| 2 | Storm | 0 *** | 0 * | 0 NS | 0 ** | 0 *** | NA |
| 5 |  | 0 NS | 0 NS | 0 * | 0 * | 0 ** | NA |
| Magnitude | | | | | | | |
| 10 | Max Month Q | 0.04 *** | 0.03 ** | 0 NS | -0.4 ** | -0.04 * | NA |
| 5 |  | 0.06 *** | 0.02 * | 0 NS | -0.16 ** | -0.01 NS | NA |
| 3 |  | 0.09 *** | 0.01 NS | 0.01 NS | -0.03 NS | 0.03 *** | NA |
| all |  | 0.07 *** | 0.03 * | 0 NS | -0.7 *** | -0.05 * | -0.6 NS |
| 10 | Min Month Q | 0.13 *** | 0.08 * | 0.01 NS | -0.64 ** | -0.06 NS | NA |
| 5 |  | 0.18 *** | 0.11 ** | -0.05 NS | -1.19 *** | -0.04 NS | NA |
| 3 |  | 0.21 *** | 0.12 * | -0.07 NS | -1.47 *** | -0.01 NS | NA |
| all |  | 0.15 *** | 0.09 * | 0.01 NS | -0.67 *** | -0.06 NS | -2.61 NS |
| 10 | Q01 | 1.16 *** | -0.09 NS | -0.15 NS | -2.05 NS | -0.1 NS | NA |
| 5 |  | 0.61 *** | 0.29 * | -0.09 NS | -1.85 ** | 0.21 NS | NA |
| 3 |  | 0.45 *** | 0.31 ** | -0.05 NS | -1.38 *** | 0.18 NS | NA |
| all |  | 2.74 *** | -3.15 *** | 6.3 NS | -1.13 NS | 0.19 NS | -2.29 NS |
| 10 | Q05 | 0.9 *** | -0.08 NS | -0.07 NS | -1.95 * | -0.29 NS | NA |
| 5 |  | 0.43 *** | 0.28 ** | -0.09 NS | -1.76 ** | 0.06 NS | NA |
| 3 |  | 0.39 *** | 0.27 ** | -0.07 NS | -1.17 *** | 0.1 NS | NA |
| all |  | 1.53 *** | -0.94 * | 0.47 NS | -0.77 NS | 0.23 NS | -1.7 NS |
| 10 | Q10 | 0.45 *** | 0.24 NS | -0.06 NS | -1.83 ** | -0.21 NS | NA |
| 5 |  | 0.32 *** | 0.24 ** | -0.07 NS | -1.17 ** | -0.01 NS | NA |
| 3 |  | 0.34 *** | 0.23 ** | -0.06 NS | -1.33 *** | 0.09 NS | NA |
| all |  | 0.79 *** | -0.17 NS | 0.07 NS | -2.8 *** | -0.02 NS | -1.54 NS |
| 10 | Q25 | 0.2 *** | 0.09 NS | -0.04 NS | -1.01 ** | -0.09 NS | NA |
| 5 |  | 0.2 *** | 0.15 ** | -0.05 NS | -1.07 *** | -0.03 NS | NA |
| 3 |  | 0.22 *** | 0.15 ** | -0.06 NS | -1.55 *** | 0.02 NS | NA |
| all |  | 0.18 *** | 0.12 ** | 0.01 NS | -1.62 *** | -0.12 NS | -1.64 NS |
| 10 | Q50 | 0.1 *** | 0.06 * | -0.02 NS | -1.13 *** | -0.05 NS | NA |
| 5 |  | 0.12 *** | 0.07 ** | -0.03 NS | -1.29 *** | -0.05 NS | NA |
| 3 |  | 0.13 *** | 0.07 * | -0.03 NS | -1.37 *** | -0.01 NS | NA |
| all |  | 0.1 *** | 0.07 ** | 0 NS | -0.87 *** | -0.06 NS | -1.43 NS |
| 10 | Q75 | 0.06 *** | 0.03 NS | -0.01 NS | -0.83 *** | -0.04 NS | NA |
| 5 |  | 0.07 *** | 0.04 * | 0 NS | -0.82 *** | -0.03 NS | NA |
| 3 |  | 0.07 *** | 0.03 * | 0 NS | -0.81 *** | -0.03 NS | NA |
| all |  | 0.06 *** | 0.03 * | 0 NS | -0.73 *** | -0.04 * | -1.21 * |
| 10 | Q90 | 0.03 *** | 0.02 ** | 0 NS | -0.32 *** | -0.03 * | NA |
| 5 |  | 0.04 *** | 0.02 ** | 0 NS | -0.31 *** | -0.02 NS | NA |
| 3 |  | 0.06 *** | 0.02 * | 0 NS | -0.19 *** | -0.01 NS | NA |
| all |  | 0.03 *** | 0.02 ** | 0 NS | -0.27 *** | -0.02 * | -0.67 * |
| 10 | Q95 | 0.02 *** | 0.01 *** | 0 NS | -0.1 *** | -0.01 * | NA |
| 5 |  | 0.03 *** | 0.02 ** | 0 NS | -0.08 ** | -0.01 NS | NA |
| 3 |  | 0.04 *** | 0.02 ** | 0 NS | -0.07 ** | 0 NS | NA |
| all |  | 0.02 *** | 0.02 *** | 0 NS | -0.11 *** | -0.01 NS | -0.23 NS |
| 10 | Q99 | 0 *** | 0 NS | 0 NS | 0 NS | 0 NS | NA |
| 5 |  | 0.01 *** | 0 NS | 0 NS | 0 NS | 0 NS | NA |
| 3 |  | 0.01 *** | 0 * | 0 NS | 0 NS | 0 * | NA |
| all |  | 0 *** | 0 NS | 0 NS | -0.01 * | 0 * | -0.01 *** |
| 10 | Qmax | 0.01 *** | 0 ** | 0 NS | -0.01 * | -0.01 ** | NA |
| 5 |  | 0.01 *** | 0 NS | 0 NS | 0 NS | 0 NS | NA |
| 3 |  | 0.01 *** | 0 NS | 0 NS | 0 NS | 0 *** | NA |
| all |  | 0.01 *** | 0 * | 0 NS | -0.06 ** | -0.01 * | -0.01 *** |
| 10 | Qmean | 0.05 *** | 0.02 * | -0.01 NS | -0.26 ** | -0.02 NS | NA |
| 5 |  | 0.07 *** | 0.03 * | -0.01 NS | -0.21 ** | -0.01 NS | NA |
| 3 |  | 0.09 *** | 0.04 * | -0.01 NS | -0.2 ** | 0.02 NS | NA |
| all |  | 0.05 *** | 0.03 * | 0 NS | -0.33 ** | -0.02 NS | -0.61 NS |
| 10 | Q mean MEDIAN | 0.06 *** | 0.04 * | 0 NS | -0.71 *** | -0.04 NS | NA |
| 5 |  | 0.08 *** | 0.04 * | 0 NS | -0.66 ** | -0.02 NS | NA |
| 3 |  | 0.16 *** | 0.03 NS | 0.01 NS | -0.18 * | 0.03 NS | NA |
| all |  | 0.07 *** | 0.03 * | 0 NS | -0.92 *** | -0.04 NS | -1.25 * |
| 10 | Qmed | 0.1 *** | 0.04 NS | -0.01 NS | -1.33 *** | -0.07 NS | NA |
| 5 |  | 0.11 *** | 0.07 * | -0.02 NS | -1.41 *** | -0.05 NS | NA |
| 3 |  | 0.12 *** | 0.07 ** | -0.01 NS | -1.25 *** | -0.04 NS | NA |
| all |  | 0.09 *** | 0.05 * | 0 NS | -0.69 *** | -0.07 NS | -2.03 * |
| 10 | Qmin | 0.16 *** | 0.1 * | 0.02 NS | -0.73 ** | -0.05 NS | NA |
| 5 |  | 0.2 *** | 0.13 ** | -0.01 NS | -0.91 ** | -0.02 NS | NA |
| 3 |  | 0.21 *** | 0.12 ** | -0.06 NS | -1.09 *** | 0 NS | NA |
| all |  | 0.17 *** | 0.13 ** | 0.03 NS | -0.88 *** | -0.03 NS | -4.87 * |
| month | Oct | 0.11 *** | 0.09 ** | -0.06 NS | -0.72 * | -0.01 NS | NA |
| month | Nov | 0.11 *** | 0.1 ** | 0.01 NS | -1.13 ** | -0.01 NS | NA |
| month | Dec | 0.16 *** | 0.07 * | 0 NS | -0.54 ** | 0 NS | NA |
| month | Jan | 0.03 *** | 0 NS | 0 NS | 0.02 NS | 0 NS | NA |
| month | Feb | 0.09 *** | 0.01 * | 0 NS | -0.04 NS | 0.01 * | NA |
| month | Mar | 0.11 *** | 0.03 ** | 0 NS | 0 NS | 0.04 ** | NA |
| month | Apr | 0.15 *** | 0.08 *** | 0 NS | -0.17 ** | 0.03 NS | NA |
| month | May | 0.18 *** | 0.06 * | -0.02 NS | -0.65 *** | 0.02 NS | NA |
| month | Jun | 0.18 *** | 0.06 * | -0.01 NS | -0.78 ** | 0.02 NS | NA |
| month | Jul | 0.2 *** | 0.06 NS | -0.02 NS | -0.83 * | 0.03 NS | NA |
| month | Aug | 0.1 *** | 0.08 ** | -0.06 * | -0.7 ** | -0.04 NS | NA |
| month | Sep | 0.1 *** | 0.08 ** | -0.05 NS | -0.71 * | -0.02 NS | NA |
| Variability | | | | | | | |
| 10 | Q min IDR | 0.1 *** | 0.02 NS | -0.03 NS | -1.2 *** | -0.05 NS | NA |
| 5 |  | 0.15 *** | 0.03 NS | -0.01 NS | -1.33 *** | -0.08 NS | NA |
| 3 |  | 0.26 *** | 0.07 NS | -0.01 NS | -1.34 ** | -0.07 NS | NA |
| all |  | 0.07 *** | 0.03 NS | 0.01 NS | -1.03 ** | -0.05 NS | -1 NS |
| 10 | Q max IDR | 0 *** | 0 NS | 0 NS | 0 NS | 0 NS | NA |
| 5 |  | 0 *** | 0 NS | 0 NS | 0 NS | 0 NS | NA |
| 3 |  | 0 *** | 0 NS | 0 NS | 0 NS | 0 NS | NA |
| all |  | 0 *** | 0 NS | 0 NS | 0 NS | 0 NS | 0 *** |
| 10 | Q mean IDR | 0.03 *** | 0.01 NS | -0.01 NS | -0.09 *** | -0.01 NS | NA |
| 5 |  | 0.04 *** | 0.01 NS | 0 NS | -0.07 * | -0.01 NS | NA |
| 3 |  | 0.08 *** | 0.03 NS | -0.01 NS | -0.09 ** | 0.03 * | NA |
| all |  | 0.03 *** | 0.01 NS | 0 NS | -0.08 *** | -0.02 * | -0.03 *** |

### **S-6:** Performance of the random forest prediction of species occurrence using streamflow metrics.

Confusion matrices show the number of successes for each species. The first table shows the accuracy of predicting presence or absence on the training data and the second shows the accuracy for the testing data. The columns across the top (blue) are the numbers based on the species observation data and the rows (tan) show the model prediction. For example, in this first table, based on the observations, 32 arroyo chub were present. The model correctly identified 30 of them and mislabeled two as absent. Based on the observations 146 locations did not have arroyo chub present, and our modeled mislabeled one as being present. (A) arroyo chub; (B) *O. mykiss irideus*; (C) Santa Ana sucker; (D) western pond turtle; (E) arroyo toad; (F) least Bell’s vireo.

(A) arroyo chub

| Training | Presence | Absence |
| --- | --- | --- |
| Presence | 30 | 1 |
| Absence | 2 | 145 |

| Testing | Presence | Absence |
| --- | --- | --- |
| Presence | 4 | 2 |
| Absence | 2 | 51 |

(B) *O. mykiss irideus*

| Training | Presence | Absence |
| --- | --- | --- |
| Presence | 78 | 0 |
| Absence | 5 | 342 |

| Testing | Presence | Absence |
| --- | --- | --- |
| Presence | 18 | 2 |
| Absence | 6 | 115 |

(C) Santa Ana sucker

| Training | Presence | Absence |
| --- | --- | --- |
| Presence | 23 | 0 |
| Absence | 0 | 163 |

| Testing | Presence | Absence |
| --- | --- | --- |
| Presence | 10 | 1 |
| Absence | 1 | 50 |

(D) western pond turtle

| Training | Presence | Absence |
| --- | --- | --- |
| Presence | 177 | 0 |
| Absence | 8 | 28 |

| Testing | Presence | Absence |
| --- | --- | --- |
| Presence | 54 | 0 |
| Absence | 2 | 15 |

(E) arroyo toad

| Training | Presence | Absence |
| --- | --- | --- |
| Presence | 18 | 0 |
| Absence | 0 | 7 |

| Testing | Presence | Absence |
| --- | --- | --- |
| Presence | 6 | 2 |
| Absence | 0 | 0 |

(F) least Bell’s vireo

| Training | Presence | Absence |
| --- | --- | --- |
| Presence | 28 | 1 |
| Absence | 0 | 20 |

| Testing | Presence | Absence |
| --- | --- | --- |
| Presence | 9 | 0 |
| Absence | 0 | 7 |

### S-7: Results of the principal components analysis.

Table S-7: Results of the principal components analysis. We used only the first two in our logistic regression to predict probability of occurrence.

| Stream temperature metric | PC1 | PC2 | PC3 | PC4 | PC5 | PC6 |
| --- | --- | --- | --- | --- | --- | --- |
| Variability explained with each principal component | | | | | | |
| Proportion of variance | 0.61 | 0.36 | 0.03 | 0.01 | 0.00 | 0.00 |
| Cumulative proportion of variance | 0.61 | 0.96 | 0.99 | 1.00 | 1.00 | 1.00 |
| Variable importance and correlation with each principal component | | | | | | |
| Max 7-Day Mean | 0.34 | -0.51 | -0.31 | 0.57 | -0.03 | -0.45 |
| Max 7-Day Max | 0.52 | -0.08 | -0.28 | 0.02 | -0.39 | 0.71 |
| Min 7-Day Min | 0.21 | -0.62 | 0.01 | -0.7 | 0.29 | -0.04 |
| Max 7-Day Range | 0.41 | 0.42 | -0.3 | 0.06 | 0.75 | 0.05 |
| Mean 7-Day Range | 0.41 | 0.41 | -0.13 | -0.4 | -0.44 | -0.54 |
| 7-Day Max > 30°C | 0.49 | 0.01 | 0.85 | 0.18 | 0.05 | 0 |

### S-8: Relationship between probability of occurrence for each species as predicted by the logistic regression with the two principal components and the temperature metrics used to create the two principal components.


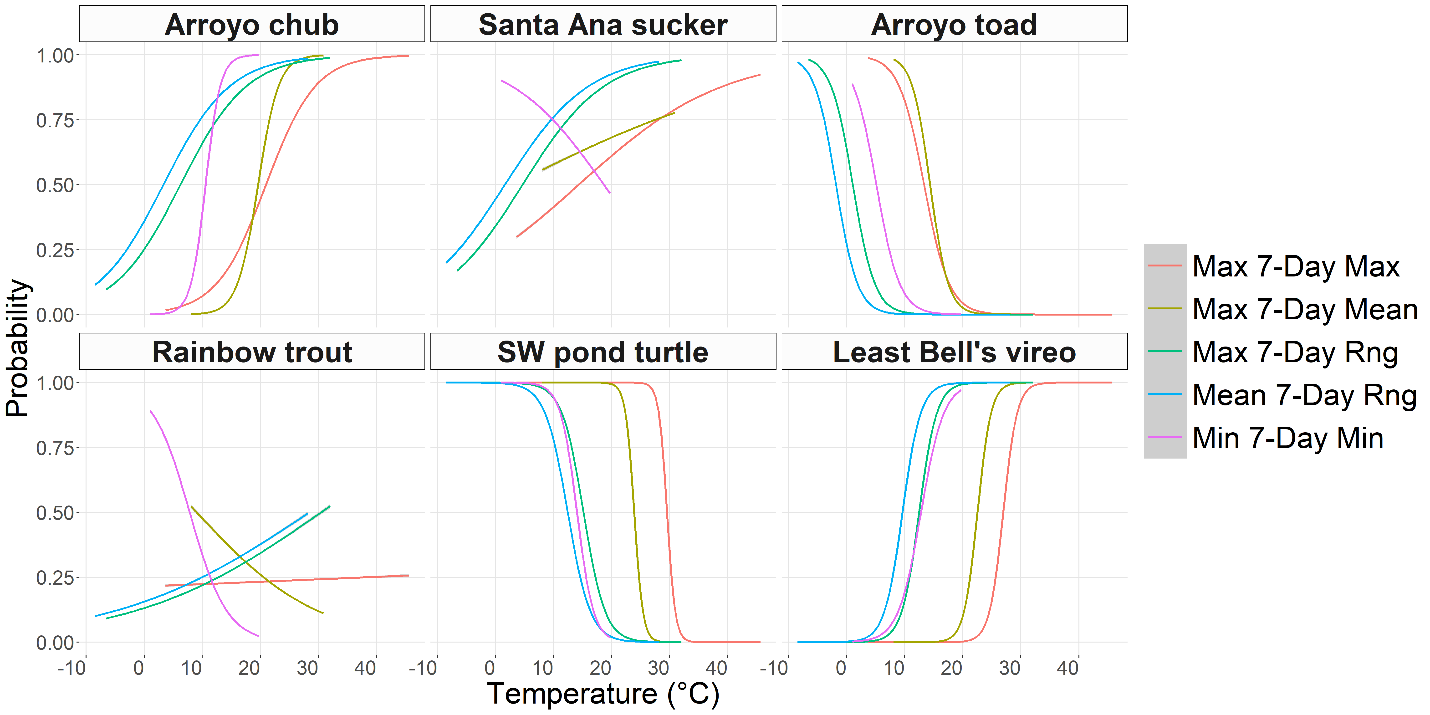


Figure S8: The relationship between the predicted probability of occurrence from the logistic regression model, which used the two principal components as predictor variables, and the original temperature metrics (in the different colors) to help interpret the relationship between stream temperature and habitat suitability. The number of days that temperatures exceed 30°C is left out because the unit is different from the other variables.
