# Supplementary material for: The impact of climate change induced alterations of streamflow and stream temperature on the distribution of riparian species
Source: PLoS One. 2020 Nov 24;15(11):e0242682. doi: 10.1371/journal.pone.0242682 (PMC7685490; doi:10.1371/journal.pone.0242682)
Supplement: Attachment — Submitted filename: PONE-D-20-20248_reviewer.pdf [file pone.0242682.s002.pdf]

PLOS ONE

The impact of climate change induced alterations of streamflow and stream temperature on the distribution of riparian species

--Manuscript Draft--

**Manuscript Number:**	PONE-D-20-20248
**Article Type:**	Research Article
**Full Title:**	The impact of climate change induced alterations of streamflow and stream temperature on the distribution of riparian species
**Short Title:**	Climate change and riparian species
**Corresponding Author:**	Jennifer B. Rogers, D. Env.<br>Southern California Coastal Water Research Project<br>Costa Mesa, California UNITED STATES
**Keywords:**	Species distribution modeling; riparian habitat; climate change; ecohydrology; stream temperature; habitat conservation; habitat suitability

Abstract:

Distributions of riparian species will likely shift due to climate change induced alterations in temperature and rainfall patterns, which alter stream habitat. Spatial forecasting of suitable habitat in projected climatic conditions will inform management interventions to help wildlife persist. Challenges in developing forecasts include the need to consider the large number of riparian species that might respond differently to changing conditions and the need to evaluate the many different characteristics of streamflow and stream temperature that drive species-specific habitat suitability. In particular, in dynamic environments like streams, the short-term temporal resolution of species occurrence and streamflow need to be considered to best understand the types of conditions that species occur in. To address these challenges, we cluster species to select habitat representatives and we evaluate regional changes in habitat suitability using short-term temporally explicit metrics that describe the streamflow and stream temperature regime. We use stream-specific environmental predictors rather than climatic variables and we use environmental data from the time of species occurrence in addition to long term trends to better evaluate habitat preferences. With species occurrence data from local monitoring surveys and streamflow and stream temperature modeled from downscaled Coupled Model Intercomparison Project - Phase 5 (CMIP5) climate projections, we predict change in habitat suitability at the end-of-century. The relative importance of hydrology and stream temperature varied by cluster. High altitudinal cold water species distributions contracted, while lower elevation, warm water species distributions expanded. Modeling with short-term temporally explicit environmental metrics did produce different end-of-century projections than using long-term averages for some of the focal species. These findings can help wildlife managers prioritize conservation efforts, manage streamflow, initiate monitoring of species in vulnerable clusters, and address stressors, such as passage barriers, in areas projected to be suitable under climate change.

"habitat representatives..." means a species that is present in that habitat and represents a group of species in a cluster?

The environmental data is the time of species occurence, or the stream-specific predictors are generated for the time when species were observed at a given reach?

"focal" is the same as "habitat representatives"? If yes, use the same term in both places.

"in future climate conditions"?

**Order of Authors:**	Jennifer B. Rogers, D. Env.
	Eric D. Stein
	Marcus W. Beck
	Richard F. Ambrose
**Additional Information:**	
**Question**	**Response**
**Financial Disclosure**<br><br>Enter a financial disclosure statement that describes the sources of funding for the work included in this submission. Review the submission guidelines for detailed	Award recipient: SCCWRP<br><br>This work was funded by the Los Angeles Regional Water Quality Control Board, Region Four under Agreement No. 16-026-140<br><br>https://www.waterboards.ca.gov/losangeles/

Powered by Editorial Manager® and ProduXion Manager® from Aries Systems Corporation

requirements. View published research articles from *PLOS ONE* for specific examples.<br><br>This statement is required for submission and **will appear in the published article** if the submission is accepted. Please make sure it is accurate.<br><br>**Unfunded studies**<br>Enter: *The author(s) received no specific funding for this work.*<br><br>**Funded studies**<br>Enter a statement with the following details:<br>• Initials of the authors who received each award<br>• Grant numbers awarded to each author<br>• The full name of each funder<br>• URL of each funder website<br>• Did the sponsors or funders play any role in the study design, data collection and analysis, decision to publish, or preparation of the manuscript?<br>• **NO** - Include this sentence at the end of your statement: *The funders had no role in study design, data collection and analysis, decision to publish, or preparation of the manuscript.*<br>• **YES** - Specify the role(s) played.<br><br>* typeset	YES: The funders had input into the study design, NO: but no role in, data collection and analysis, decision to publish, or preparation of the manuscript.
**Competing Interests**<br><br>Use the instructions below to enter a competing interest statement for this submission. On behalf of all authors, disclose any competing interests that could be perceived to bias this work—acknowledging all financial support and any other relevant financial or non-financial competing interests.<br><br>This statement **will appear in the published article** if the submission is accepted. Please make sure it is accurate. View published research articles from *PLOS ONE* for specific examples.	The authors have declared that no competing interests exist.

Powered by Editorial Manager® and ProduXion Manager® from Aries Systems Corporation

**NO authors have competing interests**<br><br>Enter: *The authors have declared that no competing interests exist.*<br><br>**Authors with competing interests**<br><br>Enter competing interest details beginning with this statement:<br><br>*I have read the journal's policy and the authors of this manuscript have the following competing interests: [insert competing interests here]*<br><br>* typeset	
**Ethics Statement**<br><br>Enter an ethics statement for this submission. This statement is required if the study involved:<br><br>• Human participants<br>• Human specimens or tissue<br>• Vertebrate animals or cephalopods<br>• Vertebrate embryos or tissues<br>• Field research<br><br>Write "N/A" if the submission does not require an ethics statement.<br><br>General guidance is provided below. Consult the submission guidelines for detailed instructions. **Make sure that all information entered here is included in the Methods section of the manuscript.**	N/A

**Format for specific study types**<br><br>**Human Subject Research (involving human participants and/or tissue)**<br>• Give the name of the institutional review board or ethics committee that approved the study<br>• Include the approval number and/or a statement indicating approval of this research<br>• Indicate the form of consent obtained (written/oral) or the reason that consent was not obtained (e.g. the data were analyzed anonymously)<br><br>**Animal Research (involving vertebrate animals, embryos or tissues)**<br>• Provide the name of the Institutional Animal Care and Use Committee (IACUC) or other relevant ethics board that reviewed the study protocol, and indicate whether they approved this research or granted a formal waiver of ethical approval<br>• Include an approval number if one was obtained<br>• If the study involved *non-human primates*, add *additional details* about animal welfare and steps taken to ameliorate suffering<br>• If anesthesia, euthanasia, or any kind of animal sacrifice is part of the study, include briefly which substances and/or methods were applied<br><br>**Field Research**<br><br>Include the following details if this study involves the collection of plant, animal, or other materials from a natural setting:<br>• Field permit number<br>• Name of the institution or relevant body that granted permission	
**Data Availability**<br><br>Authors are required to make all data underlying the findings described fully available, without restriction, and from the time of publication. PLOS allows rare exceptions to address legal and ethical concerns. See the PLOS Data Policy and FAQ for detailed information.	No - some restrictions will apply

Powered by Editorial Manager® and ProduXion Manager® from Aries Systems Corporation

A Data Availability Statement describing where the data can be found is required at submission. Your answers to this question constitute the Data Availability Statement and **will be published in the article**, if accepted.<br><br>**Important:** Stating 'data available on request from the author' is not sufficient. If your data are only available upon request, select 'No' for the first question and explain your exceptional situation in the text box.<br><br>Do the authors confirm that all data underlying the findings described in their manuscript are fully available without restriction?	
**Describe where the data may be found in full sentences. If you are copying our sample text, replace any instances of XXX with the appropriate details.**<br><br>• If the data are **held or will be held in a public repository**, include URLs, accession numbers or DOIs. If this information will only be available after acceptance, indicate this by ticking the box below. For example: *All XXX files are available from the XXX database (accession number(s) XXX, XXX.)*.<br>• If the data are all contained **within the manuscript and/or Supporting Information files**, enter the following: *All relevant data are within the manuscript and its Supporting Information files.*<br>• If neither of these applies but you are able to provide **details of access elsewhere**, with or without limitations, please do so. For example:<br><br>*Data cannot be shared publicly because of [XXX]. Data are available from the XXX Institutional Data Access / Ethics Committee (contact via XXX) for researchers who meet the criteria for access to confidential data.*<br><br>*The data underlying the results presented in the study are available from (include the name of the third party*	The data and analysis scripts are available in this repository https://github.com/JennyTay/FutureBioImpacts_Flow_Temp and archived with Zenodo here: https://zenodo.org/badge/latestdoi/253941508<br><br>Included in these repositories is all temperature and stream flow metric data for all time periods and all NHD reaches in the region. Excluded is the raw species occurrence data, which was collected by other agencies and some of which is not public. However, in the supplement we do list all the original sources of the species occurrence data. If this raw data need be made public, we would need to work with some of the agencies to determine how to do so.

and contact information or URL).<br>• This text is appropriate if the data are owned by a third party and authors do not have permission to share the data.<br><br>* typeset	
Additional data availability information:	

Powered by Editorial Manager® and ProduXion Manager® from Aries Systems Corporation

Manuscript Click here to access/download;Manuscript;manuscript.docx

The impact of climate change induced alterations of streamflow and stream temperature on the distribution of riparian species

Climate change and riparian species

Jennifer B. Rogers [1], Eric D. Stein[1], Marcus W. Beck[1,#a], Richard F. Ambrose[2]

[1] Southern California Coastal Water Research Project. 3535 Harbor Blvd., Suite 110, Costa Mesa, CA, USA 92626-1437. ORCID iD: https://orcid.org/0000-0002-4515-0753.

[2] Department of Environmental Health Sciences. Box 951772, Rm 46-078 CHS. University of California. Los Angeles, CA, USA 90095-1772

[#a] Current Address: Tampa Bay Estuary Program, 263 13th Ave South, St. Petersburg, FL, USA 33701

* Corresponding author

Email: jbtaylor339@gmail.com (JBR)

Abstract

Distributions of riparian species will likely shift due to climate change induced alterations in temperature and rainfall patterns, which alter stream habitat. Spatial forecasting of suitable habitat in projected climatic conditions will inform management interventions to help wildlife persist. Challenges in developing forecasts include the need to consider the large number of riparian species that might respond differently to changing conditions and the need to evaluate the many different characteristics of streamflow and stream temperature that drive species-specific habitat suitability. In particular, in dynamic environments like streams, the short-term temporal resolution of species occurrence and streamflow need to be considered to best understand the types of conditions that species occur in. To address these challenges, we cluster species to select habitat representatives and we evaluate regional changes in habitat suitability using short-term temporally explicit metrics that describe the streamflow and stream temperature regime. We use stream-specific environmental predictors rather than climatic variables and we use environmental data from the time of species occurrence in addition to long term trends to better evaluate habitat preferences. With species occurrence data from local monitoring surveys and streamflow and stream temperature modeled from downscaled Coupled Model Intercomparison Project - Phase 5 (CMIP5) climate projections, we predict change in habitat suitability at the end-of-century. The relative importance of hydrology and stream temperature varied by cluster. High altitudinal, cold water species distributions contracted, while lower elevation, warm water species distributions expanded. Modeling with short-term temporally explicit environmental metrics did produce different end-of-century projections than using long-term averages for some of the focal species. These findings can help wildlife managers prioritize conservation efforts, manage streamflow, initiate monitoring of species in vulnerable clusters,

and address stressors, such as passage barriers, in areas projected to be suitable under climate change.

Keywords

Species distribution modeling, riparian habitat, climate change, ecohydrology, stream temperature, habitat conservation, habitat suitability

Introduction

Climate change is expected to be a leading cause of species extinction (1,2). These concerns are particularly relevant for riparian species because of the combined effect of changing temperature and rainfall patterns, which have led to predictions of range contraction and expansion for different species (3,4). Riparian species will vary in their vulnerability to climate change depending on environmental tolerances, capacity for adaptation, geographic range size, and ability to migrate within the watershed (5). Management of riparian communities can be more effective with models that predict which species are likely to be vulnerable to changing conditions. For example, species distribution models that reveal trends in habitat availability may initiate proactive conservation projects or monitoring of species in vulnerable habitats even if the species is not currently threatened. However, developing regional projections of species response to climate change is challenging because relationships between stream condition and species' life history tolerances are rarely known except for well-studied species. The sheer number of streamflow and temperature metrics make is hard to evaluate which metrics are most critical for habitat suitability (6), the dynamic nature of both stream conditions and biology, and the presence of microclimates in many regions make it hard to draw conclusions about habitat needs from large scale or long-term averaged conditions.

The importance of considering the spatial scale of the predictor variable's has been investigated in habitat suitability modeling (7,8), however the temporal scale of predictor variables has not been explored. Species distribution modeling studies that use future projections from climate models use annual or long-term averages of environmental conditions (9–13), but the environmental averages used do not consider the interannual variation in population size or occurrence and how that relates to the interannual variation environmental conditions. For example, they do not consider that some years the species may be absent entirely, or in other years there may be lots of breeding. In stream habitats, long-term timeseries of streamflow have been used to develop predictive models for habitat suitability because they capture the regional interannual variability (3). However, attributing long-term averaged streamflow metrics to a species which breeds only after floods, for example, may misdirect efforts to predict future occurrences. In particular, for short lived aquatic species in climatically extreme areas like regions with Mediterranean climate, populations are highly dynamic in response to recent climate events and long-term averages do not describe species-specific habitat preferences that vary annually. Additionally, considering the short-term streamflow that impacts species occurrence or abundance becomes especially important considering climate models are predicting more intense and frequent extreme precipitation events (14–16) despite a similar interannual average.

There are two aims to this study and six watersheds in southern California are used as a case study. The first is to predict regional habitat suitability trends of species that represent clusters of riparian species that occur longitudinally (headwaters to estuary) and laterally (thalweg to floodplain) in the river network, in response to projected changes in streamflow and stream temperature. A focal species approach (17), which selects species to represent different

habitats, is used to evaluate impacts to biological communities that occur in similar niches. The second aim is to demonstrate an approach for habitat suitability modeling that uses environmental predictors that spatially and temporally align with the species occurrence data and therefore more accurately describe suitable habitat conditions. To accomplish these aims, we model baseline and end-of-century habitat suitability for six focal riparian species with metrics that describe the streamflow and temperature regime. We use regionally downscaled predictions for streamflow and stream temperature from CMIP5 climate modeling that is mapped to the date and location of the species occurrence or absence point, and includes short- and long-term durations of data. We model the probability of end-of-century species occurrence with projected changes in streamflow and stream temperature under climate change scenarios.

Methods

Study region

This study focused on natural and semi-natural streams in six major watersheds throughout Los Angeles and Ventura counties in southern California, southwestern United States (Fig 1). Streams in this region drain steep and geologically young mountains to the Pacific Ocean and include perennial, intermittent, and ephemeral flows. The region experiences a Mediterranean climate and receives an average of 400mm of rain annually, almost entirely during the winter months. Stream reach elevation ranges from sea level to 2646m (18) and high elevations experience snow during the winter. Common riparian shrubs and trees include species of willow, Fremont cottonwood, white alder, California sycamore, big-leaf maple, California black walnut, and mulefat (19). Federally and State endangered and threatened riparian species occur in the region and some are endemic to just a few watersheds. We limited our focus to the

mainly unaltered sub-watersheds, much of which exist in the mountains, where stream habitats and floodplains are still intact and changes in climate will be a leading impact on riparian habitat and fauna, rather than land use changes.

Fig 1. Study region with inset map showing location within California, USA, in the top left panel. The flow lines shown are included in this analysis. Blank watershed spaces are areas with heavily altered streams and not included in this analysis. Point represent the observed species data obtained for the study region. Each point represents a unique survey. Grey points denote species absence and black points denote species presence. Note: If a species observation point is shown in areas where there is no flowline, they are occurring along altered streams reaches and not included in this analysis unless a flow gauge is present in that stream reach. Flow line source: USGS national hydrography dataset (NHD).

Species selection

We selected species to model using a combined focal species approach (17) and trait-based approach (20). We selected species that occupy different niches available in the river, longitudinally (headwaters to coastal plain) and laterally (main channel to floodplain), using a clustering approach and selecting focal species from the clusters.

We identified sixty-six riparian vertebrates in the study area by reviewing regional databases and reports. We included species that require stream or riparian habitat for at least one of their life phases. Through a literature review and input from local experts, we created a life history database that described habitat and behavioral characteristics for each species that will be impacted by climate change, such as stream velocity and vegetation preference (S-1). We separated the birds from the fish, amphibians, and reptiles because the birds occupied a more terrestrial ecological niche and we did not want that difference to mask the more nuanced

differences of stream habitat preference within each group. We transformed the habitat and life history data for both groups from categorical data to a numeric dissimilarity matrix for use in clustering using the function 'daisy' from the package "cluster" (21) in R (22) with the distance metric set to "Gower" (as opposed to Manhattan or Euclidian) which calculates distance between categorical variables based on Gower distance (23). With this method, species that have identical habitat traits are characterized as identical, and as there are more dissimilar traits between species (for example a preference for fast vs slow velocity), they are classified as more dissimilar from each other. With the dissimilarity matrix, taxa were clustered using a hierarchical clustering method and we cut the dendrogram where dissimilarities were maximized between clusters. There were five clusters of birds and five clusters of fish, amphibians, and reptiles. Final clusters (S-2) were modified and ultimately two additional clusters were made based on feedback from local experts.

Of the twelve clusters that represented habitat characteristics of riparian species, we selected five clusters to model. We excluded clusters that were composed of entirely invasive species because there is no conservation concern for these clusters. Next, we excluded clusters of habitat generalists, such as dabbling ducks or herons, which use many parts of a stream. While massive changes in water availability would no doubt impact these habitat generalists, subtle changes in streamflow or temperature will have little direct impact, although we do acknowledge that their prey may be impacted by stream changes. Additionally, habitat generalists have been found to relate poorly to focal species (24). From the five remaining clusters, focal species (Table 1) were selected based on threatened or endangered listing status, the species more dependent on streams than other aquatic habitats, and for modeling purposes, species with reliable occurrence data in the study region. In one cluster, two focal species were

selected because they met all the criteria and are both of high management interest. The six focal species in order of geographic range size (largest to smallest) include Southern California steelhead/rainbow trout (*Oncorhynchus mykiss irideus*), Southwestern pond turtle (*Actinemys marmorata*), Least Bell's vireo (*Vireo bellii pusillus*) (breeding only), arroyo toad (*Anaxyrus californicus*), arroyo chub (*Gila orcuttii*), and Santa Ana sucker (*Catostomus santaanae*).

Table 1. Focal species for habitat modeling. Conservation status codes: SSC (California Species of Special Concern); FT (federally threatened); FE (federally endangered); ST (state threatened); SE (state endangered). Level of endemism codes: Low (west coast drainages of North America), Medium (central California through Baja California, Mexico), High (Los Angeles region only), CA (California).

Common name	Scientific name	Life phase	Cluster description	Conservation status	Mean (sd), and max elevation (m) of species occurrence records	Endemism level
Southern California steelhead/ rainbow trout	*Oncorhynchus mykiss irideus*	All	Cool, swift, high gradient streams, coarse substrate, deep pools	FE (Steelhead)	440(317), 1450	Low: Alaska through southern CA
Southwestern pond turtle	*Actinemys marmorata*	Juvenile / adult	Warm, low to mid gradient stream, deep pools	SSC	246(168), 711	Low: Washington to North Baja CA, Mexico

Least Bell's vireo	*Vireo bellii pusillus*	Breeding pair	Dense, 5-10 year successional stage, riparian vegetation	FE SE	173(109), 400	Medium: Central CA to Baja CA, Mexico
Arroyo toad	*Anaxyrus californicus*	Clutch	Temporary shallow backwater pools, sandy substrate	FE SSC	426(196), 1061	Medium San Luis Obispo county to Baja CA, Mexico
Arroyo chub	*Gila orcuttii*	All	Warm, sluggish, shallow, backwater or main channel, low to mid gradient streams	SSC	345(184), 1153	High: Santa Barbara to San Diego County
Santa Ana sucker	*Catostomus santaanae*	All	Warm to cool flowing water, coarse substrate, low to mid gradient stream	FT	387(162), 808	High: Los Angeles and Orange Counties

Species distribution data

We compiled the focal species observations within the study region from manuscripts, reports, standardized surveys, memos, and unpublished data sets for the years 1981 through 2017 (Fig 1); sources are shown in S-3. A limitation of this approach is the data consisted of disparate sampling efforts and are not distributed randomly across the entire region, however, Soranno[25] found that in many cases combining local data sets can be as good for prediction as data from

random sampling. Species observations were only included if location at the National Hydrography Dataset (NHD) reach scale (https://www.usgs.gov/core-science-systems/ngp/national-hydrography) and temporal information at the month scale (or finer) were reported. To minimize pseudo-replication (26), occurrences reported in the same stream reach or the same month were combined into a single data point to ensure that the analysis was not biased toward data rich streams. For example, if a surveyor reported an occurrence every 10m in a stream where they were seining for fish, we assigned that NHD stream reach a single occurrence point, not an occurrence point at each seine location within the reach.

Species occurrence was recorded as presence or absence. If a total count or abundance was provided, a single 'present' point was recorded. Absence was assumed when a surveyor failed to find the species they were looking for or when a surveyor did not record the presence of a species in certain locations but did in others. In some cases, the surveyor was contacted to ensure a lack of species record could be considered an absence rather than a lack of reporting. It is important to recognize that surveys do not achieve perfect detection (27) and thus there is greater uncertainty in the absence data than the presence data.

Environmental data

This analysis investigated the impact of streamflow and stream temperature on the distribution of species. In Mediterranean regions, like southern California, total streamflow displays large annual variation and most of the precipitation falls in a few storm events (28). Therefore, the biologically relevant aspects of the annual hydrograph are better quantified with temporally explicit streamflow metrics that describe attributes such as recession rates or timing of events compared to annual averages (29). Streamflow metrics were modeled as described in Rogers et al., (In Review) using gridded air temperature and precipitation data for three baseline

periods terminating in a representative wet (1993), dry (2014), and moderate year (2010), and three predicted end-of-century years: wet (2095), a dry (2090) and a moderate year (2100). Annual temperature metrics were also modeled as described in Rogers et al., (In Review), for baseline years (1982 – 2014) and end-of-century (2082-2100) The following section briefly describes the approach from Rogers et al., (In Review).

End-of-century streamflow and stream temperature were modeled using climate data from three downscaled global climate models (GCM's) from the Coupled Model Intercomparison Project - Phase 5 (CMIP5) ensemble to consider a range of possible future scenarios assuming the Representative Concentration Pathway (RCP) 8.5 business as usual scenario (30). The three GCM's include CanESM2, CCSM4, and MIROC5, which were among the ten highest rated models for planning in California based on historical performance and their ability to capture California's climate variability (31).

Daily flow time series were compiled either from flow gages (baseline years only) or from HEC-HMS rainfall runoff modeling (baseline and future years) of a subset of watersheds using modeled precipitation data. Hydrologic metrics (S-4) were calculated from the flow time series for each species' presence or absence record at the time and location of the biological survey. Each metric was calculated for short-~~term~~ periods from when a species occurrence was observed (3-, 5-, and 10-year) and for the entire duration of the timeseries. The hydrologic metrics were modeled regionally (i.e. for every stream reach) using a random forest approach in R (32) driven by physical basin characteristics and precipitation, resulting in a metric for each NHD stream reach for the baseline and end-of-century years. End-of-century streamflow metrics were averaged across the three GCMs.

But you modelled streamflow with HEC--why need the RF with precip?

I don't understand the reach-scale predictions of hydrolgic metric...you have observations or HEC-HMS model. Where you have observations, how do you estimate hydrologic metrics for 2090s? Where you have the HEC model, do you use HEC to "downscale" from a lumped watershed scale?

Weekly stream temperature maximum, minimum, and mean at the NHD reach scale were modeled using air temperature, watershed elevation, and watershed area. The model was trained with local water temperature gages and gridded air temperature data sets. This model was applied to end-of-century air temperature, which was downscaled from the three GCMs. Six stream temperature metrics (S-4) were calculated for each year and location with a species observation record, and for all the NHD reaches in the baseline and end-of-century time periods. End-of-century stream temperature metrics were averaged across the three GCMs.

Biological modeling

We used a species distribution modeling approach (33–35) to predict probability of species occurrence by relating the baseline streamflow and stream temperature metrics to the species occurrence data.

Streamflow metrics at all time frames (3-, 5-, 10-, and all-year) were first tested for the strength of the relationship with each focal species presence or absence using simple logistic regression (S-5). The metrics that were found to be insignificant ($P>0.05$, except for *A. californicus* where we set the cutoff to $P>0.2$ because there were so few observations) were removed from the pool of metrics. The remaining streamflow metrics were used to predict species occurrence with a random forest model, which accommodates the large number of streamflow metrics that had significant univariate relationships with each species occurrence. The model was trained with a binary ‘presence’ or ‘absence’ outcome, and we converted this to probability of species occurrence by considering the percentage of time that a ‘presence’ outcome occurred out of the 500 trees that were produced. Validation of the random forest models was done using 25% of the data that had been removed from the training data (Table 2).

Model performance shown in S-6. We used the models to predict the probability of occurrence during the baseline and the end-of-century time periods for the wet, dry, and moderate periods.

Table 2. Performance of the random forest model that used the streamflow metrics to predict species distribution. The low accuracy and high error rate of *A. californicus* is reflective of the low number of observations. The validation data accuracy is the percentage of times the model predicted correctly on the testing dataset. Error rate (out-of-bag estimate of error rate) is the percentage of observations that were misclassified in the training dataset.

	O. mykiss irideus	*A. marmorata*	*V. bellii pusillus*	*A. californicus*	*G. orcuttii*	*C. santaanae*
Validation data accuracy (%)	0.94	0.97	1.0	0.75	0.93	0.94
Error rate (%)	7.29	5.63	6.12	32	15.73	8.47

How many observations of each? Could that be included as a separate row in this table?

It seems the validation data had higher accuracy than the training data (if Error=100-accuracy). That seems surprising, no?

Logistic regression modeling was used to predict species 'presence' and 'absence' with the stream temperature metrics. We used logistic regression instead of random forest because there were far fewer temperature metrics compared to streamflow metrics. Probability of occurrence was calculated from the log odds (log(p / (1 – p))) by: $P(occurrence) = \exp(\text{log odds})/(1 + \exp(\text{log odds}))$. To minimize collinearity between the six stream temperature metrics, we used principal component analysis (PCA) to reduce the six variables to two uncorrelated predictors, which explained 84% of the variance of the original six metrics (S-7). We applied the PCA on the standardized baseline and end-of-century stream temperature

metrics to calculate the principal components used in the model to calculate species occurrence annually through the end-of-century.

Temporally explicit predictor variables

To investigate the impact of using short-term temporally specific streamflow metrics, versus long-term average streamflow values in habitat suitability modeling, we modeled probability of species occurrence using two additional random forest models. In the first model, we used only the all-year flow metrics in the baseline years to predict probability of occurrence. In the second model (the short-term temporally explicit model), we used only the 3-year metrics in the baseline years to predict the probability of occurrence. We could not do this comparison for *A. marmorata* because we only had all-year baseline metrics for that species due to a lack of species observation data within the timespan of the baseline streamflow data. Both models predicted probability of species occurrence by considering the percentage of time that a ‘presence’ outcome occurred out of the 500 trees that were produced in the random forest, as described above. Models were trained with 75% of the data for each species and were assessed using the error rate and the accuracy on the remaining 25% validation data. We used the two models to predict the probability of occurrence during the end-of-century time period for the wet and dry years. We were interested both in the model performance differences and the differences between the two projected species distribution maps.

Results

Environmental Drivers

Streamflow variables that relate to stream flashiness such as RBI, recession rates, and flow durations were most associated with the of probability of species occurrence (S-5).

Oncorhynchus mykiss irideus was associated with consistent flows, i.e. there was a negative relationship with storm events and drought events, and a positive association with hydroperiod. *Anaxyrus californicus* was associated with no flow periods in the short term (3 and 5-year), but consistent flows in the stream reach over longer time periods (all-year). *Gila orcuttii* and *C. santaanae* were associated with flashiness, rapid recessions, storm events, and were negatively associated with hydroperiod (i.e. they occur in streams that do not flow for part of the year). *Gila orcuttii* was generally positively associated with streamflows at all timeframes and flow magnitudes whereas *C. santaanae* had negative relationships with flow magnitude at many of the timeframes and flow magnitudes. *Vireo bellii pusillus* had a positive relationship with flashy streams, fast recessions, and the frequency and recency of storms at all timeframes, but had a negative relationship with hydroperiod. *Vireo bellii pusillus* consistently had a negative relationship with flow magnitude. *Actinemys marmorata* was positively related to RBI, yet otherwise was associated with low flow magnitudes, a long duration of low flows, and low storm frequency.

Stream temperature was significantly related to the distribution of five of the six species although the relationships differed between species (Table 3). Relationships observed demonstrate the importance of warm and cool water habitat throughout the region. Because the results of the regression are hard to interpret with the principal components, we show the trend line for probability of occurrence that was predicted from the regression and each stream temperature metric in S-8. *Oncorhynchus mykiss irideus* and *A. californicus* consistently show a negative relationship with stream temperature for all the metrics. While the regression with *A. californicus* did not have significant results, which is expected due to the very small sample size compared to the other species, the relationship is supported by the occurrence records which

show observations in cooler water than the other species (maximum of 28.41°C, compared to the other species' maximum temperatures which ranged from 31.73 - 34.10 °C). Conversely, *G. orcuttii* and *V. bellii pusillus* have a positive relationship with stream temperatures across all metrics. While *V. bellii pusillus* is not an aquatic bird, the strong preference for warm water could be mediated through the low elevations that support their preferred riparian vegetation for nesting, their insect prey relationship with stream temperature, or some other factor associated with warm water. The turtle *A. marmorata* had a positive relationship with maximum and minimum temperatures, but a negative relationship with mean temperatures. *Catastomus santaanae* had a positive relationship with minimum and mean stream temperatures but showed almost no relationship with maximum temperatures.

Table 3. Logistic regression results from the species distribution model driven by stream temperature. * $P<0.05$, ** $P<0.01$, *** $P<0.001$. For better interpretation of the coefficients, see S-7 to see which stream temperature metrics are described by each principal component and see S-8, which shows the relationship between the probability of occurrence and each temperature metric implicit in the principal components.

Species	Variable	Coefficient (log odds)	Std. error (log odds)	P-value
O. mykiss irideus	PC 1	-0.07277	0.09917	0.46
	PC 2	-1.22944	0.15719	5.23e-15***
A. marmorata	PC 1	-0.7155	0.1965	0.0003***
	PC 2	-0.5681	0.4297	0.19
V. bellii pusillus	PC 1	0.1697	0.2798	0.54
	PC 2	2.1998	0.3487	2.81e-10***
A. californicus	PC 1	0.5213	0.6804	0.44
	PC 2	-0.7739	1.0007	0.44

G. orcuttii	PC 1	-0.3590	0.1266	0.005**
	PC 2	1.3426	0.2152	4.38e-10***
C. santaanae	PC 1	0.4163	0.2125	0.05
	PC 2	0.5851	0.2442	0.02*

Projected changes in species occurrence due to stream temperature

Regional probability of occurrence increased annually for three species and decreased annually for three species from the baseline to the end-of-century (Fig 2). By end-of-century, probability of occurrence increased most rapidly for *V. bellii pusillus* (β_0 = 0.006, R^2 = 0.77), followed by *G. orcuttii* (β_0 = 0.003, R^2 = 0.74), and finally *C. santaanae* (β_0 =0.002, R^2 = 0.67). Probability of occurrence decreased for *O. mykiss irideus* (β_0 = -0.004, R^2 = 0.72), *A. californicus* (β_0 = -0.002, R^2 = 0.74), and *A. marmorata* (β_0 = -0.002, R^2 = 0.58).

Fig 2. Annual predicted probability of occurrence from the baseline years to the end-of-century due to stream temperature changes for each species. The dark black line shows the mean probability of occurrence annually across all the stream reaches. The upper and lower light grey lines show the 95th and 5th percentile probabilities for the stream reaches. Breaks in line show the years that were missing from our analysis. The trend line is a linear model of the mean annual probability of occurrence.

We predict that species distributions will shift between high and low elevations under future climate (Fig 3). Probably of occurrence increased more for *G. orcuttii* at high vs. low elevations (mean probability of 0.35 in high elevations vs. 0.12 in low elevations; $P< 0.001$). Probability of occurrence for *C. santaanae* increased by a mean of 0.19 in high elevations vs. 0.13 in low elevations ($P< 0.001$). In contrast, probability of occurrence for *V. bellii pusillus*

increased by a mean of 0.62 in low elevations vs. mean of 0.53 in high elevations ($P < 0.001$). The other three species, which decreased in probability, all decreased more in high elevations compared to low elevations. Probability of *O. mykiss irideus* occurrence decreased by a mean of -0.37 in high elevations compared to -0.25 in low elevations ($P < 0.001$). Probability of *A. californicus* occurrence decreased by a mean of -0.24 in high elevations compared to -0.07 in low elevations ($P < 0.001$). Finally, probability of *A. marmorata* occurrence decreased by a mean of -0.19 in high elevations and -0.09 in low elevations ($P < 0.001$).

Fig 3. Change in probability of occurrence due to stream temperature. Change was calculated as the average values for each stream reach in the end-of-century minus the average values for each stream reach in the baseline period. A-F show the spatial distribution of the change. Note that the color scale is different for each species. G-L show the distribution of the change in probabilities for the stream reaches between high and low elevations, which is defined as 375m and higher. Again, note that the y-axis limits are different for each species.

Projected changes in species occurrence due to streamflow

A positive change in probability of occurrence was generally observed in the wet, moderate, and dry years for the three fish species (Table 4). Conversely, there was a negative change for the three year types for *V. bellii pusillus*. Probability of occurrence of *A. californicus* had a mean increase in the dry and moderate year, but a mean decrease in the wet year. *Actinemys marmorata* showed no change in probability of occurrence because in both time periods there were predicted probabilities of 1.0.

Table 4. The average change probability of occurrence throughout the region due to streamflow metrics from the baseline years to the end-of-century years. Change calculated

as probability of occurrence in the end-of-century minus probability of occurrence in the baseline. Numbers in parentheses show the 5th and 95th percentile.

	Dry	Moderate	Wet
Arroyo chub	0.17 (-0.05,0.39)	0.16 (-0.05,0.34)	0.11 (-0.04,0.25)
Santa Ana sucker	0.08 (-0.11,0.31)	0.05 (-0.14,0.23)	0.07 (-0.07,0.24)
Rainbow trout	0.11 (-0.05,0.29)	0.07 (-0.16,0.3)	0.12 (-0.07,0.33)
Least Bell's vireo	-0.08 (-0.2,0.04)	-0.06 (-0.18,0.06)	-0.03 (-0.12,0.08)
Arroyo toad	0.13 (-0.06,0.34)	0.04 (-0.07,0.18)	-0.02 (-0.12,0.13)
SW pond turtle	0 (0,0)	0 (0,0)	0 (0,0)

The elevation of the stream reach did impact the change in probability of occurrence in the end-of-century compared to baseline (Fig 4). The affect was manifested in two ways. First, there was a shift in the same direction but of different magnitude, as seen with *G. orcuttii*, *O. mykiss irideus*, and *V. bellii pusillus*. Second, there was a shift in the opposite direction depending on elevation. For example, probability of occurrence decreased for *C. santaanae* in low elevations but increased in high elevations. The same was observed for the *A. californicus*.

Fig 4. The distribution of probability of occurrence for the regional stream reaches due to streamflow in the baseline (light grey) and the end-of-century (dark grey) for low (<375m) and high (>375m) elevation streams. Table 4 shows the change in the different water year types. Boxplots show the 25th and 75th percentiles, and 1.5*the inter-quartile range at the upper and lower end. Points beyond the 'whiskers' represent outliers.

Impacts of stream temperature versus streamflow

Using our projections for all six species we estimate that probability of occurrence in end-of-century compared to baseline is likely going to be driven more by changes in stream temperature than streamflow (Fig 5). Averaging across year types, the average change in probability of occurrence of *G. orcuttii* due to flow was 0.15 vs. 0.30 due to temperature ($P < 0.001$). Similarly, the average change due to streamflow and temperature was 0.06 and 0.16 (*C. santaanae*, $P < 0.001$); 0.05 and -0.21 (*A. californicus*, $P < 0.001$); 0.10 and -0.34 (*O. mykiss irideus*, $P < 0.001$); 0.00 and -0.15 (*A. marmorata*, $P < 0.001$); and -0.06 and 0.54 (*V. bellii pusillus*, $P < 0.001$), respectively. In all cases, the change due to stream temperature exceeds the change due to streamflow.

Fig 5. The distribution of probability of occurrence for the regional stream reaches due to streamflow (light grey) and stream temperature (dark grey) in the baseline and end-of-century time period (future). In each time period, the lower distribution would be considered the variable that is limiting habitat suitability for each species. Boxplots show the 25th and 75th percentiles, and 1.5*the inter-quartile range at the upper and lower end. Points beyond the 'whiskers' represent outliers.

The limiting variable stayed the same in the baseline and end-of-century for four species and changed for two. For example, in the baseline year, temperature was limiting for *A. californicus* and temperature continued to be limiting in the end-of-century. Conversely, in the baseline year streamflow was limiting for *O. mykiss irideus*, but stream temperature became limiting in the end-of-century.

Temporally explicit predictor variables

The 3-year predictor model had an error rate less than or equal to the error rate for the all-year predictor model (Table 5). Validation data accuracy was the same between the two models except for two species, where each model had a higher accuracy. Probability of occurrence predictions differed between the two models (Fig 6). The temporally explicit model predicted higher probability of occurrence across the study region for *G. orcuttii*, *C. santaanae, O. mykiss irideus*, but lower probability of occurrence for *A. californicus* and *V. bellii pusillus*. In general, the relative predicted species distribution across the region were similar between the models, despite the difference in magnitudes, except for *A. californicus* where the temporally explicit model interestingly predicted lower probability of occurrence in the high elevation streams compared to the low elevation streams, whereas the all-year model predicted higher probabilities in the high elevation streams compared to the low elevation streams.

Table 5. Comparison of the two random forest models that used the all-year vs 3-year predictor variables. Timeframe refers to the model that was used. All means long-term averaged streamflow metrics were used in the model using the entire duration of streamflow timeseries. Three means that only short-term streamflow data was used in training the model. For example, only 3 years of streamflow data, that terminated on the date of species observation, were used to calculate the streamflow metrics. The validation data accuracy is the percentage of times the model predicted correctly on the testing dataset. Error rate (out-of-bag estimate of error rate) is the percentage of observations that were misclassified in the training dataset.

	O. mykiss irideus		*V. bellii pusillus*		*A. californicus*		*G. orcuttii*		*C. santaanae*	
Timeframe	All	3	All	3	All	3	All	3	All	3
Validation data accuracy (%)	0.89	0.94	1.0	1.0	0.63	0.63	0.97	0.92	0.97	0.97
Error rate (%)	11.06	7.53	8.16	8.16	28.0	24.0	15.17	12.92	5.91	4.30

Figure 6. End-of-century probability of occurrence predictions using the all-year predictors and the 3-year predictors. The first column shows the predicted end-of-century probabilities using streamflow data from a long-term timeseries including the entire timeseries of forecasted years. The middle column shows predicted end-of-century probability in a three year dry period, and the last column shows predicted end-of-century probability in a three year wet period.

Discussion

Species vulnerability patterns

Two characteristics emerged as potential drivers of vulnerability that could be applied regionally to other species in the clusters: Species that occur in high elevation stream habitat and a small environmental niche, were more vulnerable to climate change... regardless of the geographical extent of their range.

We observed that species in high elevation streams appear to be vulnerable to climate change, while those that occur in lower elevation streams either benefit or are unaffected by

predicted conditions. Alternatively, vulnerable and resilient species occurred laterally across the channel in habitats such as the main channel, shallow edge waters, deep pools, and floodplain. This suggests that longitudinal stream position may be a useful first assessment of vulnerability.

Because longitudinal position correlates with elevation? Or maybe you meant lateral?

In response to climate change, the two species that generally occur highest in the watersheds (*O. mykiss irideus* and *A. californicus*) are projected to lose suitable habitat due to increasing stream temperatures. Conversely, the species that mostly occur in low elevations (*G. orcuttii* and *V. bellii pusillus*) are projected to gain suitable habitat. An exception is *A. marmorata*, which does occur in low elevation streams but is projected to lose suitable habitat due to increasing stream temperatures. Other studies have found greater reduction in range size and risk of extinction for species in high elevation areas (36,37). Thuiller[38] found species that occur in cool high elevation areas are potentially more vulnerable toward warming, which supports our finding that *O. mykiss irideus* and *A. californicus* are projected to be temperature limited. As temperatures rise, the lower elevational limit increases in altitude (39,40), decreasing habitat at moderate elevations which historically supported the species. Thuiller[38] found that species in very warm regions are less sensitive to warming and predicted that their range expands. Therefore, a replacement of high elevational species by lower elevational species could occur at the transition between habitat types on the mountains, reducing the suitable habitat range of species occupying the highest elevations.

A decrease in habitat suitability at moderate elevations, which leaves just the highest elevations of a species range suitable, may start a negative feedback loop for additional habitat loss. Rivers are connected from headwaters to estuary and the anadromous life form of *O. mykiss irideus* migrate this length. A loss in suitability at moderate elevations could stifle recolonization by adults migrating up the watershed for spawning. Kaylor[41] found that trout

populations in the highest headwaters, unlike the other streams in their study, did not rebound after a drought which they attributed to lack of connectivity at the upstream and downstream end of the reach. In our study, we cannot determine if the decrease in habitat suitability at the downstream end of a suitable reach would stifle trout migration (in which case connectivity would be impacted), because our *O. mykiss irideus* occurrence data combined spawning and migration observations. However, it is possible that the highest headwaters become isolated from the lower watersheds which would stifle recolonization for *O. mykiss irideus*, and potentially *A. californicus* depending on their migration ability.

Surprisingly, our results showed that highly endemic species (*G. orcuttii* and *C. santaanae*) were projected to have increased habitat suitability, whereas the species with larger ranges throughout western North America (*O. mykiss irideus* and *A. marmorata*) were predicted to have decreased habitat suitability. This finding is similar to Thuiller[42] who found endemic plants in Namibia were generally more resilient to climate change than widespread ones. This could suggest that a large geographic range is associated with a negative response to climate change. However, most studies of historic and future climate change have alternatively found that endemic species were vulnerable compared to widespread species (see review: Isaac[43]). Historically, studies of climate warming during the Quaternary found that widespread species were able to inhabit regions of Europe that had previously been frozen but that are currently suitable, whereas range restricted species to this day are limited to the areas that were not frozen for reptiles and amphibians (44) and trees (45). One explanation for this is that range restricted species are limited by dispersal and therefore cannot move into new territories as they become suitable (46). This suggests that species with narrow geographic ranges may be more vulnerable than species with wider ranges because the former cannot disperse into new areas and will be

sensitive to new climate conditions in the areas they currently inhabit. We did not account for dispersal limitations because our study area is limited to six adjacent watersheds above all major dams, so, in general, dispersal, including the potential for assisted migration should be feasible. The assumption that any habitat within the region is accessible may be one reason why we did not see greater vulnerability of highly endemic species. Like the historical studies of climate warming, when predicting the relative impact of *future* climate change, Zhang[47] found species with the smallest geographic ranges to be the most vulnerable. Similarly, in a meta-analysis of 131 studies, Urban[1] found that endemic species were more vulnerable to rising temperatures and showed higher extinction risk.

However, perhaps our finding is not contradictory with these other studies of range size and climate change vulnerability. However, for each focal species, whether the full geographic range is widespread or highly endemic, we projected the habitat generalists to respond more favorably than habitat specialists (also observed by Thuiller[38]; Clavel[48]; Poff[5]), which suggests a decoupling of geographic range size and environmental niche breadth, also shown in a study of *Gammarus* spp. (49) and a study of rattlesnakes (50). While many studies have found a positive association between environmental niche breadth and geographic range (51), in our study, the species with the smallest geographic range often have relatively wide environmental niche breadths. For example, although *A. californicus* has a larger geographic range than *G. orcuttii*, within its geographic range it occupies fewer habitat types. Therefore, despite high endemicity of *G. orcuttii*, their projected response to climate change is positive, possibly due to their wide environmental niche breadth within our study region. Overall, the lack of dispersal limitation associated with endemics in this small study area, combined with the wide environmental niche breath, and warm stream temperature preferences of the highly endemic species in this study

could explain why they were less vulnerable to climate change than the more widely dispersed species.

Species conservation

Projecting the impacts of climate change on the future distributions of focal riparian species can help managers foresee vulnerabilities for even the less studied species. For example, the regions' two additional native riverine fish, unarmored threespine stickleback (*Gasterosteus aculeatus williamsoni*) and Santa Ana speckled dace (*Rhinichthys osculus* ssp.) occur in the clusters with *G. orcuttii* and *O. mykiss irideus*, respectively. Application of our results could suggest that projected streamflow patterns are not harmful for those two species. However, increasing stream temperatures likely harm *R. osculus* ssp., as extrapolated from the results for *O. mykiss irideus*. Alternatively, rising stream temperatures may support *G. aculeatus williamsoni*, as extrapolated from the results for *G. orcuttii*. *Vireo bellii pusillus* was selected as the focal species that represents a cluster of riparian nesting birds, such as yellow warbler (*Setophaga petechia*). Extrapolation of results to these other species would suggest that while projected changes in streamflow will negatively impact their nesting habitat, increases in stream temperature will be supportive. While using the focal species approach is not a complete substitute for species or community level study (52), it can help identify vulnerabilities and inform the direction of future studies.

Habitats suitability modeling considering the dynamic trends of species distributions improved habitat prioritization for conservation planning (53). We found that considering temporally specific environmental drivers also produced different results from the model that used long-term averages as predictor variables. For most species the locations of high and low habitat suitability did not differ between the two models despite differences in magnitude, but for

A. californicus the highest probability locations were reversed depending on the model. This suggests that habitat suitability assessment that considers interannual variation can identify conditions that support species that would be missed by using only regional averages, particularly in dynamic habitats like streams in Mediterranean climate regions.

The differences for *A. californicus*, compared to the other focal species, could have been magnified because that species in particular limits usage of the stream habitat to years that are suitable. While drought reduces the number of breeding pairs (54), Miller[61] found that alternating drought and storms supported *A. californicus* because *A. californicus* can limit breeding to years with suitable conditions while excluding predators that require permanent surface water, such as the American bullfrog (*Lithobates catesbeianus*). However, drought conditions beyond the lifespan of *A. californicus*, 6-8 years, impedes breeding opportunities and leads to significant reductions in the population (56). Temporally specific metrics can reveal this dynamic of needing surface water frequently enough to support breeding, but not consistently enough to support predators.

With these projections, conservation projects can be planned proactively (57). For example, enhancing stream connectivity between high and low elevation populations will allow the lower population to migrate before conditions become unsuitable. Additionally, the spatial prioritization of location and types of land parcels for protection (58), decisions regarding a species vulnerability and sensitivity listing, and water management decisions can be informed by predicting spatial habitat suitability. Projections of where suitable habitat conditions persist can help managers target conservation and restoration projects in watersheds that maintain those favorable conditions (59). For example, managers can begin monitoring in locations projected to

support species to assess for the presence of other stressors and opportunities to enhance the habitat such as riparian tree planting programs to buffer against rising air temperatures.

Consideration for futures studies

There are other drivers of species distribution besides streamflow and stream temperature that may change with climate change, such as biotic competition (60), barriers to dispersal (60,61), and land use (62). We did not include the entire range of some of the species which underestimates the range of environmental tolerances (63). This may underestimate impacts of climate change for species toward the southern end of their range (*O. mykiss irideus* and *A. marmorata*) and potentially overestimate impacts of climate change for species at the mid- to northern end of their range (*A. californicus*). Similarly, climate change impacts could affect *V. bellii pusillus* in other parts of their range or during migration that we could not consider (64).

Another consideration would be to model different life phases of the focal species separately. We combined the life history forms of *O. mykiss irideus*, and all aquatic life history stages of each focal species. We did this so we could include survey data when life history stage was not known. We therefore lumped all reports of either life history form of *O. mykiss irideus* and all life history stages of each focal species into the presence and absence data set, even though life history stages are associated with different environmental conditions (41,65,66) because we judged data representing as much of the study region as possible to be more important. The magnitude of the projected impact on *O. mykiss irideus* was likely diminished because we included migration observations in the species occurrence data set from low elevations, as opposed to only including observations from their spawning grounds in high elevation tributaries.

We assumed full migration capacity of all species in the future scenarios, meaning any new suitable habitat is considered regardless of proximity to the current range. Other studies have used two extreme dispersal scenarios, full or none, to determine future suitable habitat (36,67,68), however, we considered stream reaches that would be suitable for species occurrence to give wildlife managers flexibility for conservation planning. Forecasts of suitable stream reaches that are separated from the current range by natural or anthropogenic barriers can help direct assisted migration programs or connectivity design (69) and in some cases, a separate watershed may provide better refuge due to practical reasons such as less recreational use.

Conclusion

Here we have demonstrated an approach for projecting the impacts of climate change on riparian species due to changes in streamflow and stream temperature. By using temporally and spatially referenced streamflow and stream temperature metrics that align with species occurrence, models more accurately describe the conditions associated with species occurrence, which can help managers. By using species that represented different portions of the watershed, we can estimate impacts on other species with similar habitat preferences. We find that while projections differed between species, there were some patterns ~~that revealed themselves~~ that could be applied elsewhere to support an initial estimate at species vulnerabilities. Species in high altitudinal regions are likely more vulnerable to climate change. We recommend investigating the importance of short-term stream conditions for species that use the stream selectively to better model habitat suitability. Finally, we recommend this method to provide a preliminary estimation of the biological impacts of climate change that can be broadly applied to other species in the region.

Acknowledgements

We thank Alex Hall, Jerry H-Y. Huang, and Neil Berg, at the University of California, Los Angeles, Department of Atmospheric and Oceanic Sciences, for providing their downscaled air temperature and precipitation model output for baseline conditions and future projections. We thank Rosi Dagit from the Resource Conservation District of Santa Monica Mountains, Mary Larson and Jeff Weaver from California Department of Fish and Wildlife, Chris Medak and Chris Dellith from the United States Fish and Wildlife Service, and Johnathan Baskin from California State Polytechnic University Pomona for providing species occurrence data. We thank Kelly Flint and Alicia Kinoshita from the Department of Civil Engineering, San Diego State University for modeling streamflow. Finally, we thank members of our technical advisory committee for their thoughtful comments throughout the study.

References

1. Urban MC. Accelerating extinction risk from climate change. Science (80-). 2015;348(6234):571–3.

2. Thomas CD, Cameron A, Green RE, Bakkenes M, Beaumont LJ, Collingham YC, et al. Extinction risk from climate change. Nature. 2004;427:145–8.

3. Bond N, Thomson J, Reich P, Stein J. Using species distribution models to infer potential climate change-induced range shifts of freshwater fish in south-eastern Australia. Mar Freshw Res. 2011;62(9):1043–61.

4. Wenger SJ, Isaak DJ, Luce CH, Neville HM, Fausch KD, Dunham JB, et al. Flow regime, temperature, and biotic interactions drive differential declines of trout species under climate change. Proc Natl Acad Sci [Internet]. 2011;108(34):14175–80. Available from: http://www.pnas.org/cgi/doi/10.1073/pnas.1103097108

5. Poff NL, Olden JD, Strayer DL. Saving a million species: extinction risk from climate change. Hannah L, editor. Chapter 17: Climate change and freshwater fauna extinction risk. Island Press; 2012. 309–336 p.

6. Carlisle DM, Grantham TE, Eng K, Wolock DM. Biological relevance of streamflow metrics: Regional and national perspectives. Freshw Sci. 2017;36(4):927–40.

7. Fernandes JA, Rutterford L, Simpson SD, Butenschön M, Frölicher TL, Yool A, et al. Can we project changes in fish abundance and distribution in response to climate? Glob Chang Biol. 2020;(February):1–15.

8. Seavy NE, Viers JH, Wood JK. Riparian bird response to vegetation structure: a

multiscale analysis using LiDAR measurements of canopy height. Ecol Appl. 2009;19(7):1848–57.

9. Hof C, Araújo MB, Jetz W, Rahbek C. Additive threats from pathogens, climate and land-use change for global amphibian diversity. Nature. 2011;480(7378):516–9.

10. Ochoa-Ochoa LM, Rodríguez P, Mora F, Flores-Villela O, Whittaker RJ. Climate change and amphibian diversity patterns in Mexico. Biol Conserv [Internet]. 2012;150(1):94–102. Available from: http://dx.doi.org/10.1016/j.biocon.2012.03.010

11. Remya K, Ramachandran A, Jayakumar S. Predicting the current and future suitable habitat distribution of Myristica dactyloides Gaertn. using MaxEnt model in the Eastern Ghats, India. Ecol Eng [Internet]. 2015;82:184–8. Available from: http://dx.doi.org/10.1016/j.ecoleng.2015.04.053

12. Wright AN, Schwartz MW, Hijmans RJ, Bradley Shaffer H. Advances in climate models from CMIP3 to CMIP5 do not change predictions of future habitat suitability for California reptiles and amphibians. Clim Change. 2016;134(4):579–91.

13. Domisch S, Jähnig SC, Haase P. Climate-change winners and losers: Stream macroinvertebrates of a submontane region in Central Europe. Freshw Biol. 2011;56(10):2009–20.

14. Polade SD, Gershunov A, Cayan DR, Dettinger MD, Pierce, David W. Precipitation in a warming world : Assessing projected hydro-climate changes in California and other Mediterranean climate regions. Nat Sci Reports. 2017;7(February):1–10.

15. Gershunov A, Shulgina T, Clemesha RES, Guirguis K, Pierce DW, Dettinger MD, et al.

Precipitation regime change in Western North America: The role of Atmospheric Rivers. Nat Sci Reports. 2019;1–11.

16. Das T, Maurer EP, Pierce DW, Dettinger MD, Cayan DR. Increases in flood magnitudes in California under warming climates. J Hydology. 2013;501:101–10.

17. Lambeck R. Focal species: a multiple-species umbrella for nature conservation. Conserv Biol [Internet]. 1997;11(4):849–56. Available from: http://onlinelibrary.wiley.com/doi/10.1046/j.1523-1739.1997.96319.x/abstract

18. Hill RA, Weber MH, Leibowitz SG, Olsen AR, Thornbrugh DJ. The Stream-Catchment (StreamCat) Dataset: A Database of Watershed Metrics for the Conterminous United States. J Am Water Resour Assoc. 2016;52:120–8.

19. Faber PM, Keller E, Sands A, Massey BM. The Ecology of Riparian Habitats of the Southern California Coastal Region: A Community Profile. 1989.

20. Olden JD, Poff NL, Bestgen KR. Trait synergisms and the rarity, extirpation, and extinction risk of desert fishes. Ecology. 2008;89(3):847–56.

21. Maechler M, Rousseeuw P, Struyf A, Hubert M, Hornik K. cluster: Cluster Analysis Basics and Extensions. R package version 2.0.6. 2017;

22. R Core Team. R: A language and environment for statistical computing. R Foundation for Statistical Computing, Vienna, Austria. URL https://www.R-project.org/. 2019.

23. Gower JC. A General Coefficient of Similarity and Some of Its Properties. 1971;27(4):857–71.

24. Suter W, Graf RF, Hess R. Capercaillie (Tetrao urogallus) and avian biodiversity: Testing

the umbrella-species concept. Conserv Biol. 2002;16(3):778–88.

25. Soranno PA, Cheruvelil KS, Liu B, Wang Q, Tan PN, Zhou J, et al. Ecological prediction at macroscales using big data: Does sampling design matter? Ecol Appl. 2020;0(0):1–13.

26. Hurlbert SH. Pseudoreplication and the Design of Ecological Field Experiments Stuart. Ecol Monogr. 1984;54(2):40–8.

27. Lahoz-Monfort JJ, Guillera-Arroita G, Wintle BA. Imperfect detection impacts the performance of species distribution models. Glob Ecol Biogeogr. 2014;23(4):504–15.

28. Gasith A, Resh VH. Streams in mediterranean climate regions: Abiotic influences and biotic responses to predictable seasonal events. Annu Rev Ecol Evol Syst. 1999;30:51–81.

29. Yarnell SM, Petts GE, Schmidt JC, Whipple AA, Beller EE, Dahm CN, et al. Functional Flows in Modified Riverscapes: Hydrographs, Habitats and Opportunities. Bioscience. 2015;65(10):963–72.

30. IPCC. Climate Change 2014 Synthesis Report. Contribution of Working Groups I, II and III to the Fifth Assessment Report of the Intergovernmental Panel on Climate Change. [Core Writing Team, R.K. Pachauri and L.A. Meyer (eds.)]. Geneva, Switzerland; 2014.

31. DWR. Perspectives and Guidance for Climate Change Analysis. 2015.

32. Liaw A, Wiener M. Classification and Regression by randomForest. R News. 2002;2(3):18–22.

33. Araújo MB, Anderson RP, Barbosa AM, Beale CM, Dormann CF, Early R, et al. Standards for distribution models in biodiversity assessments. Sci Adv. 2019;5(1):1–12.

34. Elith J, Leathwick JR. Species Distribution Models: Ecological Explanation and

Prediction Across Space and Time. Annu Rev Ecol Evol Syst [Internet]. 2009;40(1):677–97. Available from: http://www.annualreviews.org/doi/10.1146/annurev.ecolsys.110308.120159

35. Guisan A, Zimmermann NE. Predictive habitat distribution models in ecology. Ecol Modell. 2000;135(2–3):147–86.

36. Loarie SR, Carter BE, Hayhoe K, Mcmahon S, Moe R, Knight, Charles A, et al. Climate Change and the Future of California's Endemic Flora. PLoS One. 2008;3(6):e2502.

37. Bomhard B, Richardson DM, Donaldson JS, Hughes GO, Midgley GF, Raimondo, Domitilla C, et al. Potential impacts of future land use and climate change on the Red List status of the Proteaceae in the Cape Floristic Region , South Africa. Glob Chang Biol. 2005;11:1452–68.

38. Thuiller W, Lavorel S, Araújo MB. Niche properties and geographical extent as predictors of species sensitivity to climate change. Glob Ecol Biogeogr. 2005;14(4):347–57.

39. Wilson RJ, Gutiérrez D, Gutiérrez J, Martínez D, Agudo R, Monserrat VJ. Changes to the elevational limits and extent of species ranges associated with climate change. Ecol Lett. 2005;8(11):1138–46.

40. Dirnböck T, Essl F, Rabitsch W. Disproportional risk for habitat loss of high-altitude endemic species under climate change. Glob Chang Biol. 2011;17(2):990–6.

41. Kaylor MJ, Verwey BJ, Cortes A, Warren DR. Drought impacts to trout and salamanders in cool, forested headwater ecosystems in the western Cascade Mountains, OR. Hydrobiologia [Internet]. 2019;833(1):65–80. Available from:

https://doi.org/10.1007/s10750-019-3882-2

42. Thuiller W, Midgley GF, Hughes GO, Bomhard B, Drew G, Rutherford MC, et al. Endemic species and ecosystem sensitivity to climate change in Namibia. Glob Chang Biol. 2006;12:759–776.

43. Isaac JL. Effects of climate change on life history: Implications for extinction risk in mammals. Endanger Species Res. 2009;7(2):115–23.

44. Araújo MB, Nogués-Bravo D, Diniz-Filho JAF, Haywood AM, Valdes PJ, Rahbek C. Quaternary climate changes explain diversity among reptiles and amphibians. Ecography (Cop). 2008;31(1):8–15.

45. Svenning JC, Skov F. Ice age legacies in the geographical distribution of tree species richness in Europe. Glob Ecol Biogeogr. 2007;16(2):234–45.

46. Graham CH, Moritz C, Williams SE. Habitat history improves prediction of biodiversity in rainforest fauna. Proc Natl Acad Sci U S A. 2006;103(3):632–6.

47. Zhang J, Nielsen SE, Chen Y, Georges D, Qin Y, Wang S, et al. Extinction risk of North American seed plants elevated by climate and land-use change. J Appl Ecol. 2017;54:303–12.

48. Clavel J, Julliard R, Devictor V. Worldwide decline of specialist species: Toward a global functional homogenization? Front Ecol Environ. 2011;9(4):222–8.

49. Gaston KJ, Spicer JI. The relationship between range size and niche breadth: A test using five species of Gammarus (Amphipoda). Glob Ecol Biogeogr. 2001;10(2):179–88.

50. Waldron JL, Bennett SH, Welch SM, Dorcas ME, Lanham JD, Kalinowsky W. Habitat

specificity and home-range size as attributes of species vulnerability to extinction: A case study using sympatric rattlesnakes. Anim Conserv. 2006;9(4):414–20.

51. Slatyer RA, Hirst M, Sexton JP. Niche breadth predicts geographical range size: A general ecological pattern. Ecol Lett. 2013;16(8):1104–14.

52. Simberloff D. Flagships, umbrellas, and keystones: is single-species management passe in the landscape era? Biol Conserv. 1998;83(3):247–57.

53. Johnston A, Auer T, Fink D, Strimas-Mackey M, Iliff M, Rosenberg K V., et al. Comparing abundance distributions and range maps in spatial conservation planning for migratory species. Ecol Appl. 2020;0(0):1–13.

54. Jennings MR, Hayes MP. Amphibian and Reptile Species of Special Concern in California. Rancho Cordova, CA, 95701; 1994.

55. Miller DAW, Brehme CS, Hines JE, Nichols JD, Fisher RN. Joint estimation of habitat dynamics and species interactions : disturbance reduces co-occurrence of non-native predators with an endangered toad. J Anim Ecol. 2012;1–10.

56. Fisher RN, Brehme CS, Hathaway SA, Hovey TE, Warburton ML, Stokes DC. Longevity and population age structure of the arroyo southwestern toad (Anaxyrus californicus) with drought implications. Ecol Evol. 2018;8(12):6124–6132.

57. Palmer MA, Lettenmaier DP, Poff NL, Postel SL, Richter B, Warner R. Climate change and river ecosystems: Protection and adaptation options. Environ Manage. 2009;44(6):1053–68.

58. Jones KR, Watson JE., Possingham HP, Klein CJ. Incorporating climate change into

spatial conservation prioritisation: A review. Biol Conserv [Internet]. 2016;194:121–30. Available from: http://dx.doi.org/10.1016/j.biocon.2015.12.008

59. Loyola RD, Lemes P, Nabout JC, Trindade-Filho J, Sagnori MD, Dobrovolski R, et al. A straightforward conceptual approach for evaluating spatial conservation priorities under climate change. Biodivers Conserv. 2013;22(2):483–95.

60. Davis AJ, Jenkinson LS, Lawton JH, Shorrocks B, Wood S. Making mistakes when predicting shifts in species range in response to global warming. Nature. 1998;391(February):783–6.

61. Araújo MB, Pearson RG. Equilibrium of species distributions with climate. Ecography (Cop). 2005;28(5):693–5.

62. Maloney KO, Krause KP, Buchanan C, Hay LE, Mccabe GJ, Smith ZM, et al. Disentangling the potential effects of land-use and climate change on stream conditions. Glob Chang Biol. 2020;(November 2019):1–19.

63. Sinclair SJ, White MD, Newell GR. How useful are species distribution models for managing biodiversity under future climates? Ecol Soc. 2010;15(1).

64. Zurell D, Graham CH, Gallien L, Thuiller W, Zimmermann NE. Long-distance migratory birds threatened by multiple independent risks from global change. Nat Clim Chang [Internet]. 2018;8(November):992–996. Available from: http://dx.doi.org/10.1038/s41558-018-0312-9

65. Welsh HHJ, Hodgson GR, Harvey BC, Roche MF. Distribution of Juvenile Coho Salmon in Relation to Water Temperatures in Tributaries of the Mattole River , California. North

Am J Fish Manag. 2001;21(3):464–70.

66. Spina AP. Thermal ecology of juvenile steelhead in a warm-water environment. Environ Biol Fishes. 2007;80:23–34.

67. Thuiller W, Midgley GF, Hughes GO, Bomhard B, Drew G, Rutherford MC, et al. Endemic species and ecosystem sensitivity to climate change in Namibia. Glob Chang Biol. 2006;12(5):759–76.

68. Gibson L, McNeill A, Tores P de, Wayne A, Yates C. Will future climate change threaten a range restricted endemic species, the quokka (Setonix brachyurus), in south west Australia? Biol Conserv [Internet]. 2010;143(11):2453–61. Available from: http://dx.doi.org/10.1016/j.biocon.2010.06.011

69. Loss SR, Terwilliger LA, Peterson AC. Assisted colonization: Integrating conservation strategies in the face of climate change. Biol Conserv [Internet]. 2011;144(1):92–100. Available from: http://dx.doi.org/10.1016/j.biocon.2010.11.016

Supporting Information

S-1 Table: Variables used in clustering analysis.

S-2 Table: Species clusters. Riparian species (vertebrates) in the study area that were included in the clustering analysis. The last column, ‘Cluster’, shows the grouping. The bolded rows are the species selected as the focal species

S-3: Sources used for compiling the species distribution data

S-4 Table: Streamflow and temperature metrics. Streamflow and temperature metrics. Script used for calculation and definition came from Konrad et al., 2008 except for the last three variables in the table which we calculated separately. Timeframe refers to the number of years of flow data used in the calculation, measured back in time, from the year of analysis. Bolded rows show the variables that we present in this paper. For temperature, the months included in calculating these metrics are May through September.

S-5 Table: Streamflow metrics and focal species presence or absence relationship simple logistic regression results. Univariate logistic regression results for species presence or absence and a subset of the streamflow metrics. Sign refers to the direction of the coefficient. Significance codes: $p<0.001$ ***; $p<0.01$ **; $p<0.05$ * except for A. californicus where $P<0.2$ *. The time refers to the length of streamflow timeseries used to calculate the flow metric. All = the entire streamflow timeseries; 3, 5, 10 = three, five, and ten years of flow data terminating on the date of species occurrence record. Variables defined in S-4.

S-6 Table: Performance of the random forest prediction of species occurrence using streamflow metrics. Confusion matrices show the number of successes for each species. The first table shows the accuracy of predicting presence or absence on the training data and the

second shows the accuracy for the testing data. The columns across the top (blue) are the numbers based on the species observation data and the rows (tan) show the model prediction. For example, in this first table, based on the observations, 32 arroyo chub were present. The model correctly identified 30 of them and mislabeled two as absent. Based on the observations 146 locations did not have arroyo chub present, and our modeled mislabeled one as being present. (A) arroyo chub; (B) O. mykiss irideus; (C) Santa Ana sucker; (D) western pond turtle; (E) arroyo toad; (F) least Bell's vireo.

S-7 Table: Results of the principal components analysis. Results of the principal components analysis. We used only the first two in our logistic regression to predict probability of occurrence.

S-8 Figure: Relationship between probability of occurrence for each species as predicted by the logistic regression with the two principal components and the temperature metrics used to create the two principal components. The relationship between the predicted probability of occurrence from the logistic regression model, which used the two principal components as predictor variables, and the original temperature metrics (in the different colors) to help interpret the relationship between stream temperature and habitat suitability. The number of days that temperatures exceed 30°C is left out because the unit is different from the other variables.

Figure

Click here to access/download;Figure;fig1.tiff

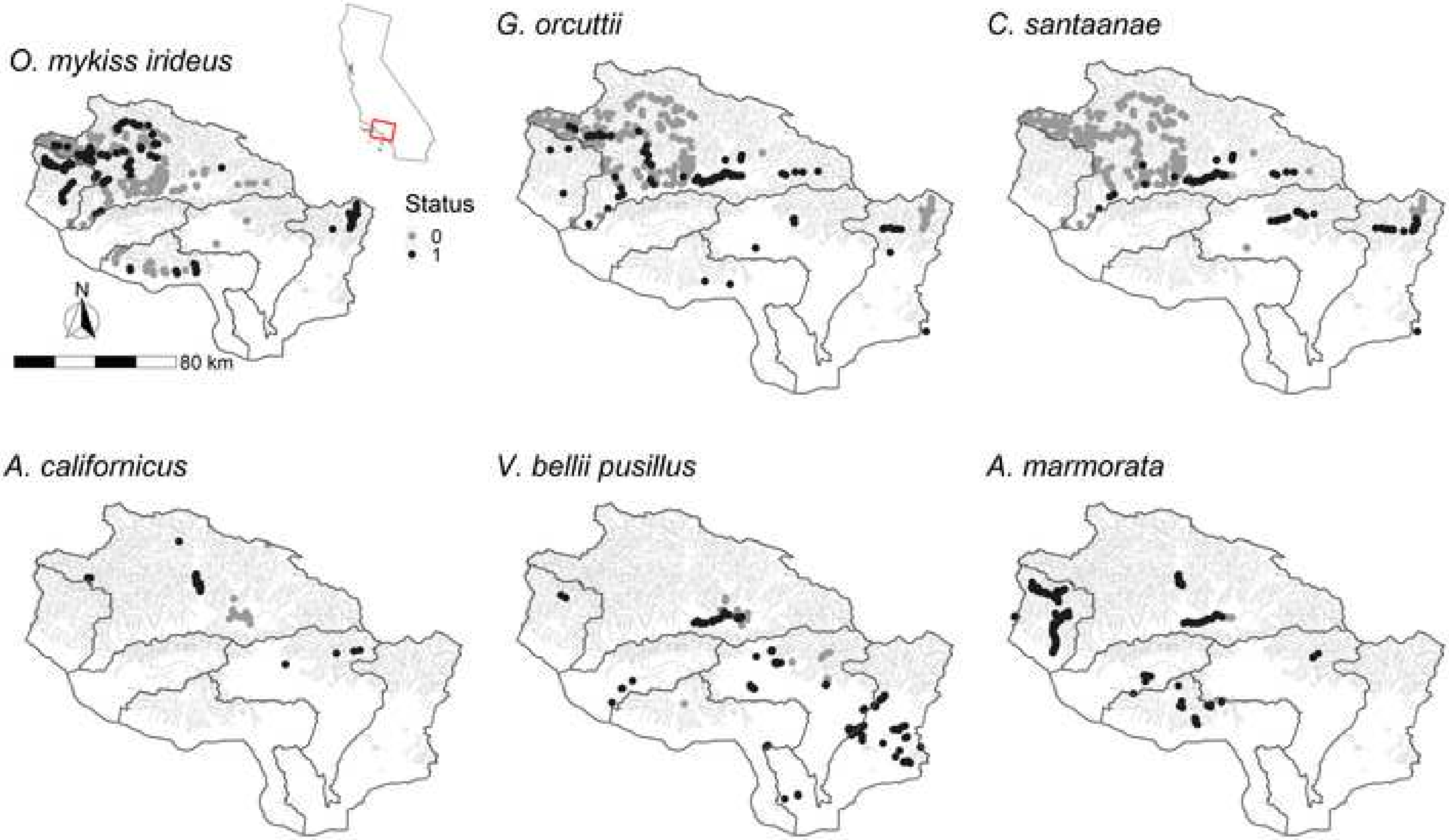

Figure

Click here to access/download;Figure;fig2.tiff

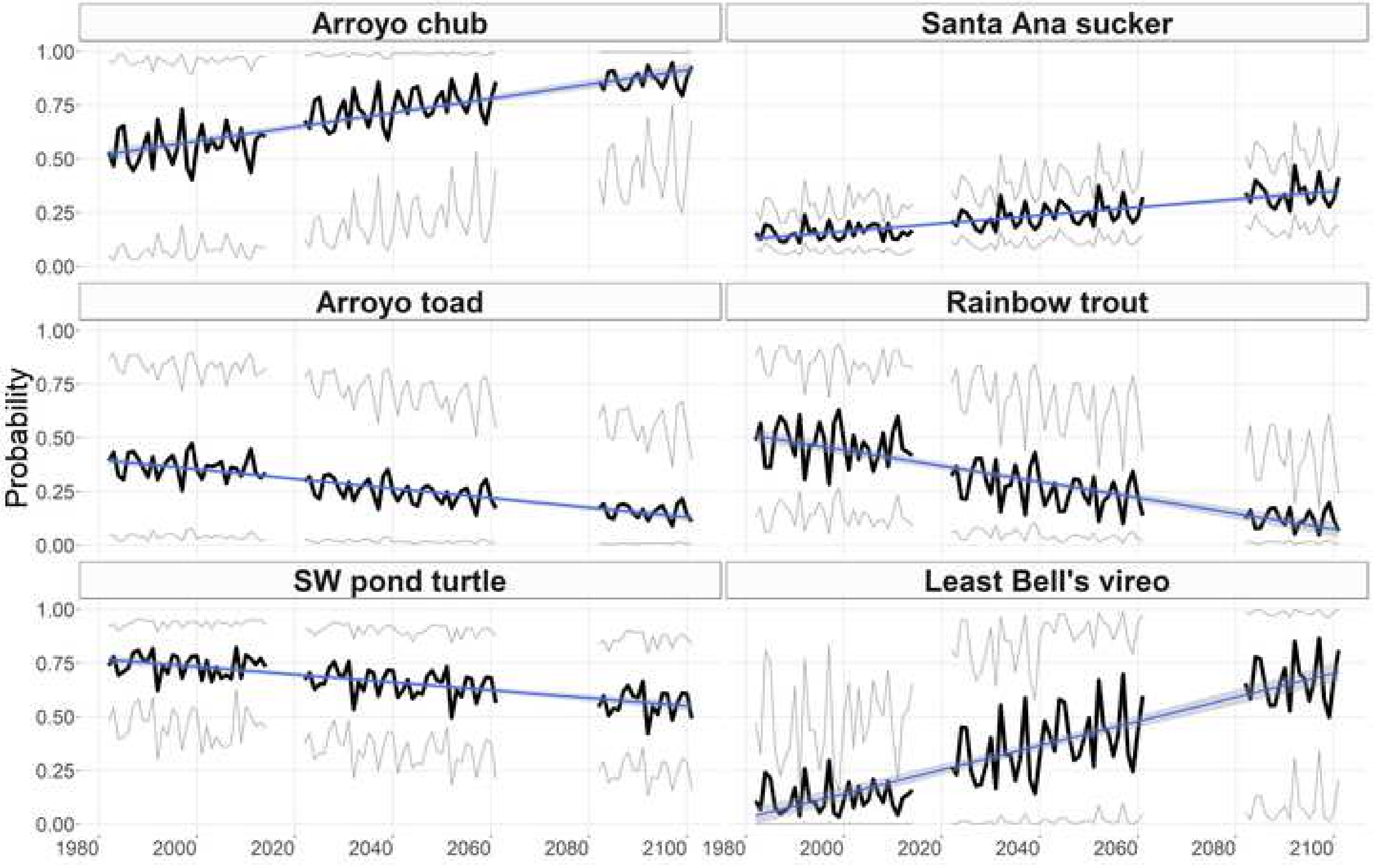

Figure

Click here to access/download;Figure;fig3.tiff

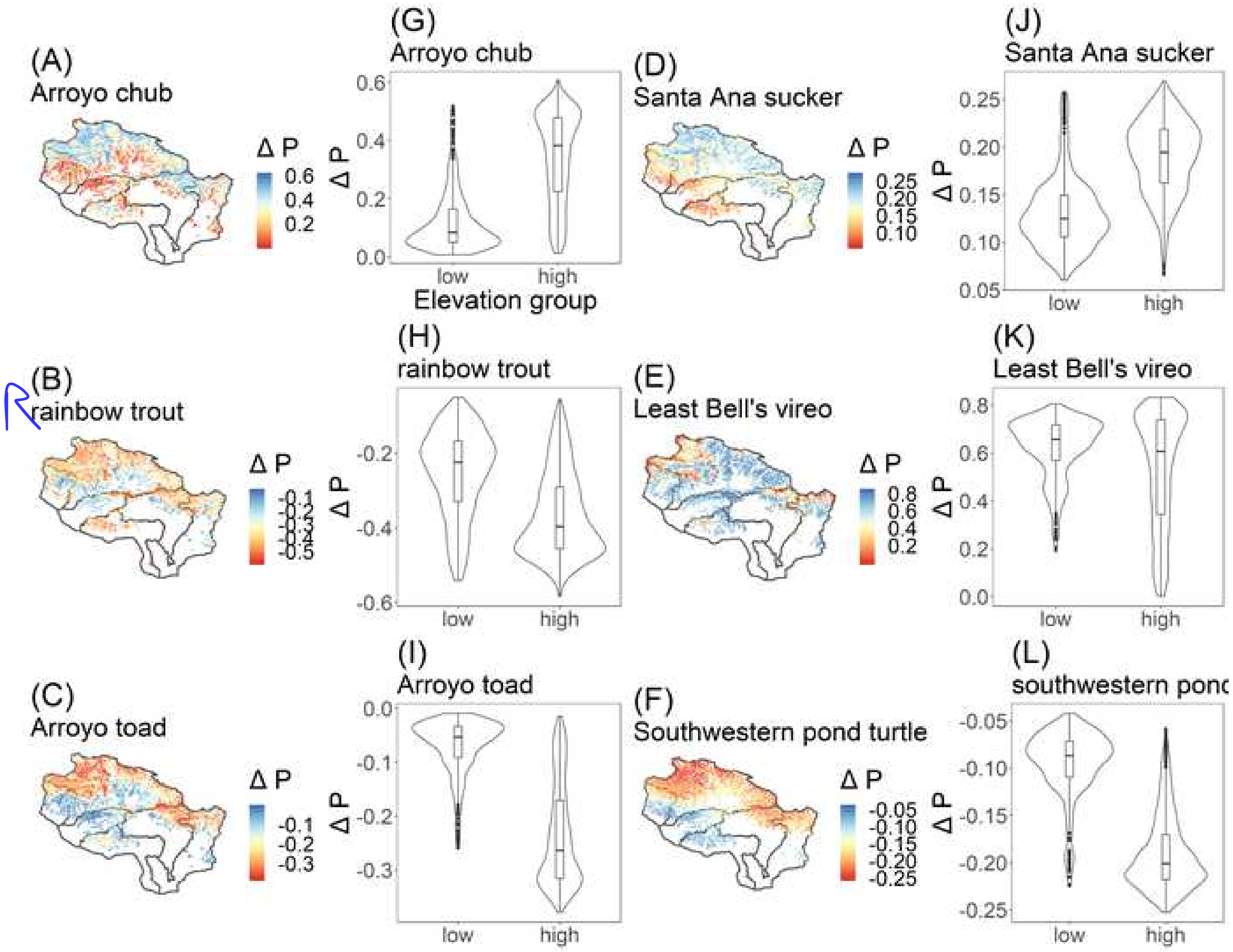

Click here to access/download;Figure;fig4.tiff

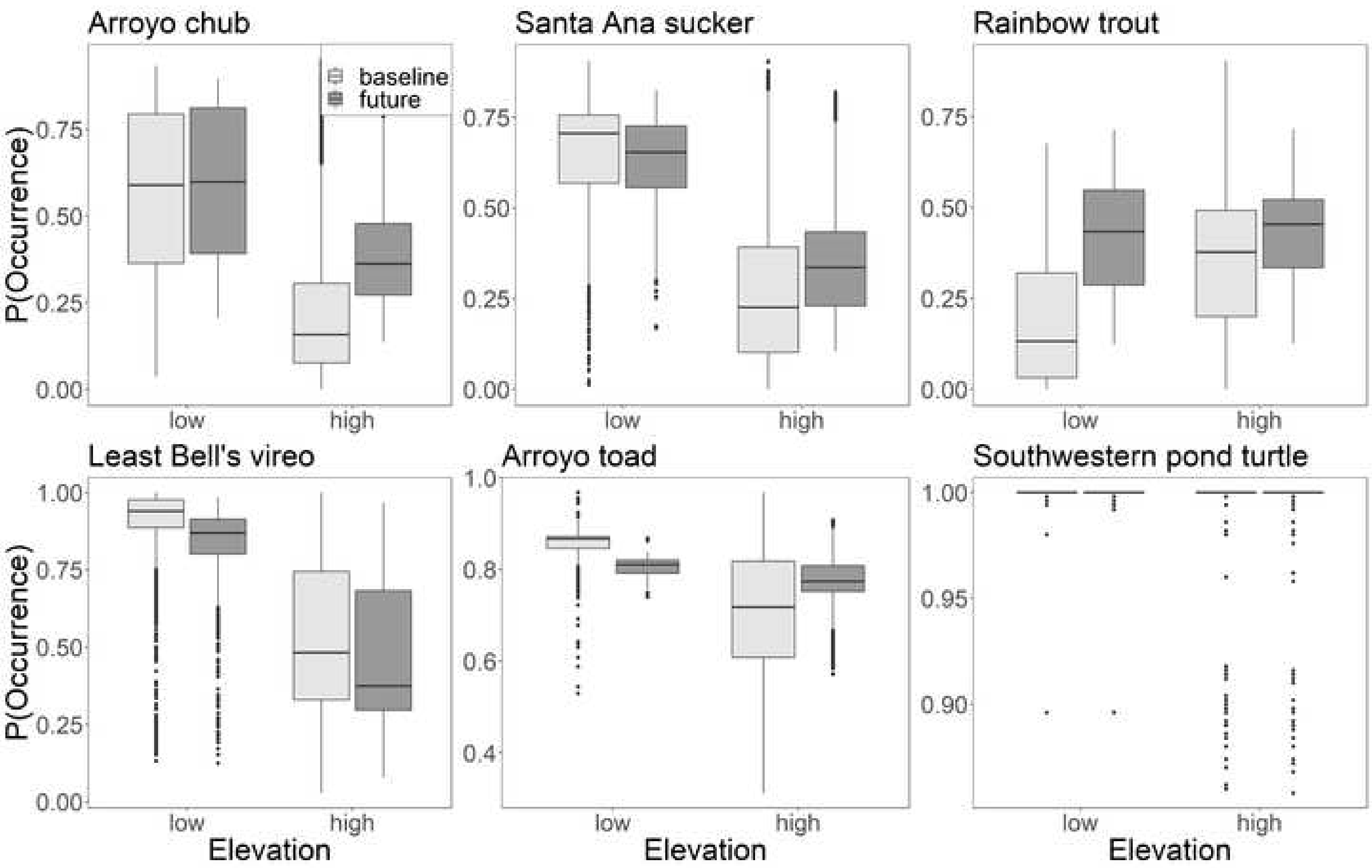

Click here to access/download;Figure;fig5.tiff

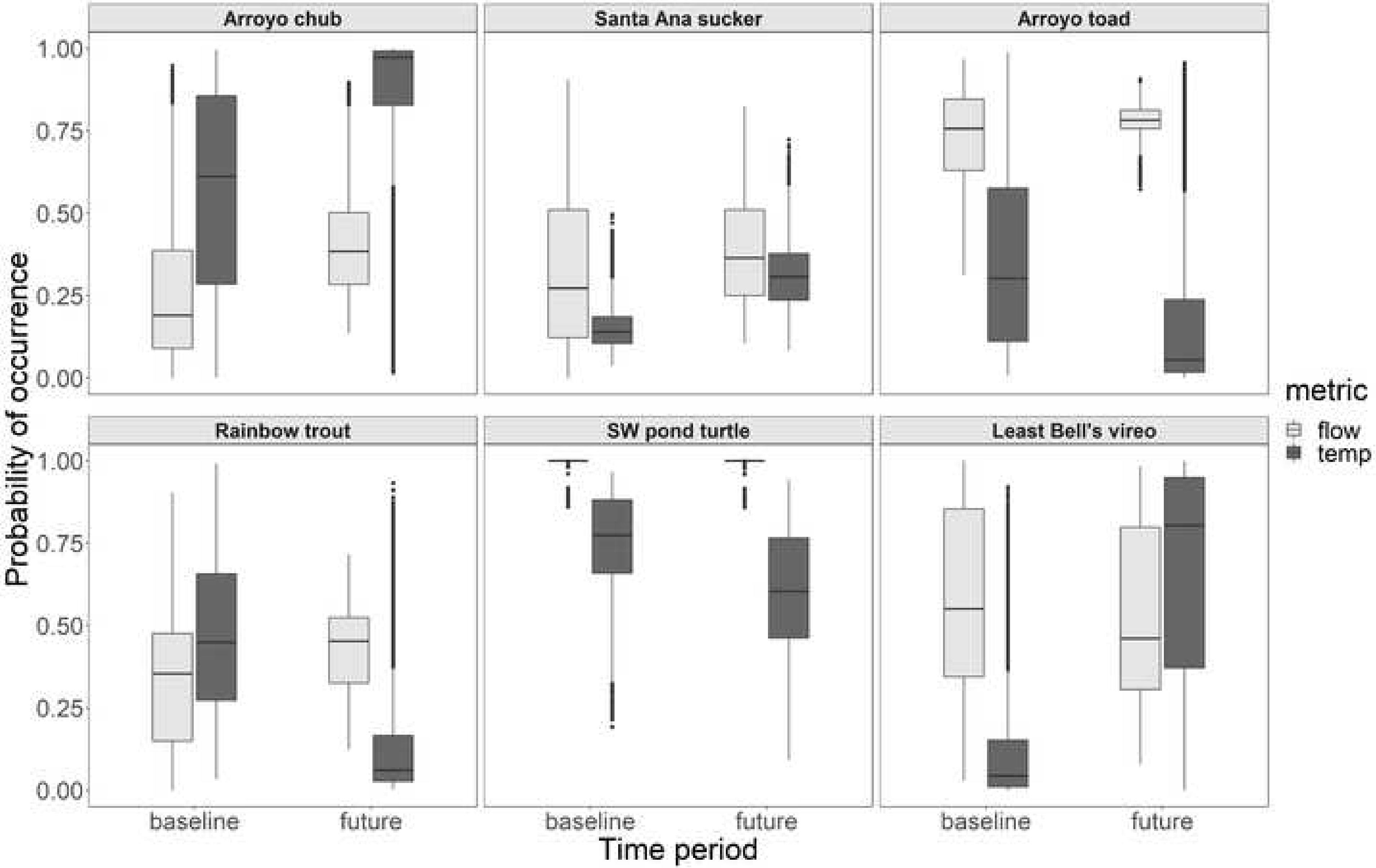

Click here to access/download;Figure;fig6.tiff

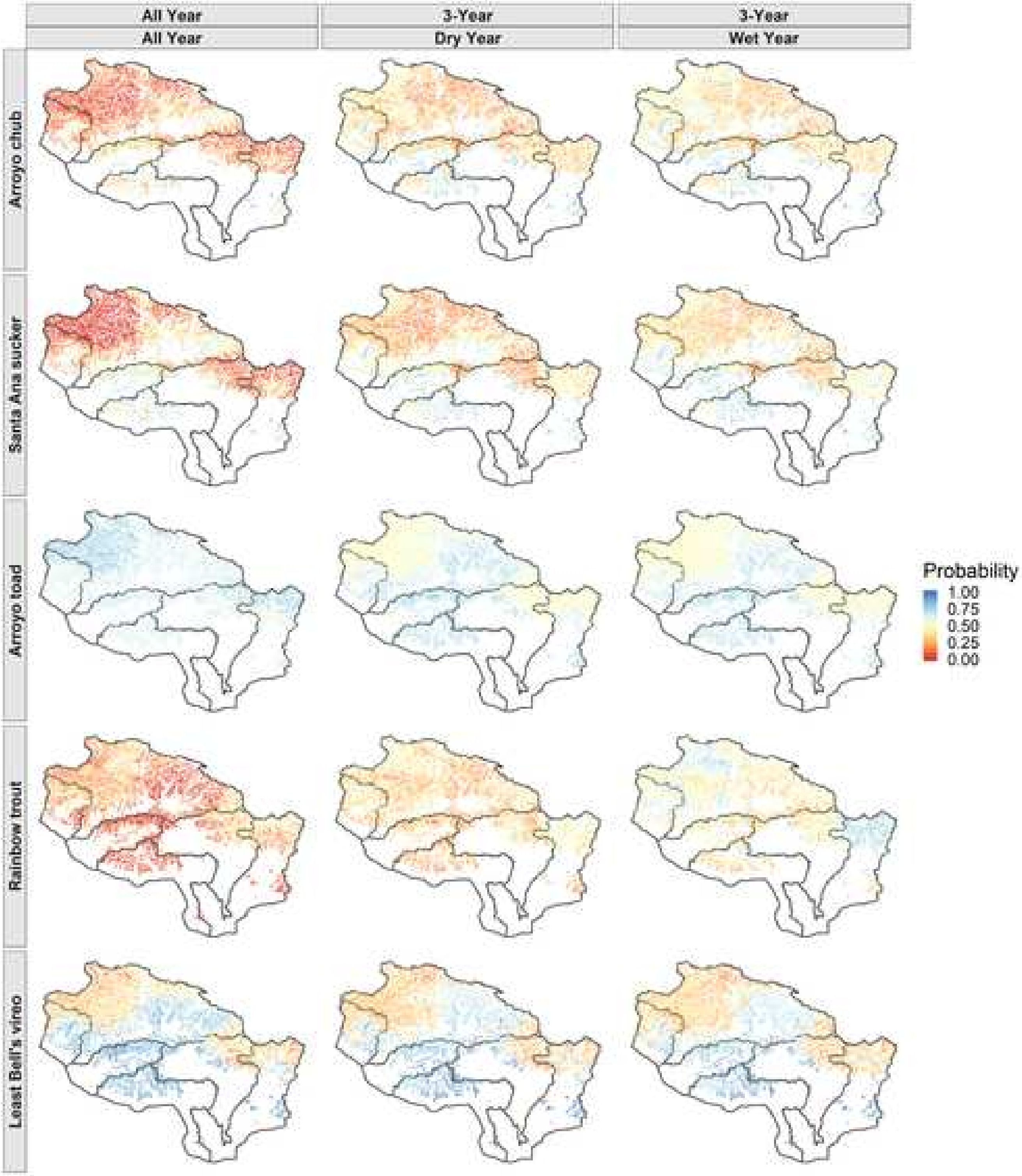

Click here to access/download
Supporting Information
SupportingInformation.docx

Data Review URL

Click here to download Data Review URL
https://zenodo.org/badge/latestdoi/253941508
